# Geographical Latitude Remains as an Important Factor for the Prevalence of Some Myositis Autoantibodies: A Systematic Review

**DOI:** 10.3389/fimmu.2021.672008

**Published:** 2021-04-22

**Authors:** Andrea Aguilar-Vazquez, Efrain Chavarria-Avila, Oscar Pizano-Martinez, Alejandra Ramos-Hernandez, Lilia Andrade-Ortega, Edy-David Rubio-Arellano, Monica Vazquez-Del Mercado

**Affiliations:** ^1^ Centro Universitario de Ciencias de la Salud, Doctorado en Ciencias Biomédicas, Universidad de Guadalajara, Guadalajara, Mexico; ^2^ Centro Universitario de Ciencias de la Salud, Instituto de Investigación en Reumatología y del Sistema Músculo-Esquelético (IIRSME), Universidad de Guadalajara, Guadalajara, Mexico; ^3^ Centro Universitario de Ciencias de la Salud, Departamento de Disciplinas Filosófico, Metodológicas e Instrumentales, Universidad de Guadalajara, Guadalajara, Mexico; ^4^ Hospital Civil Dr. Juan I. Menchaca, División de Medicina Interna, Servicio de Reumatología 004086, PNPC CONACyT, Guadalajara, Mexico; ^5^ Centro Universitario de Ciencias de la Salud, UDG-CA 703 Inmunología y Reumatología, Universidad de Guadalajara, Guadalajara, Mexico; ^6^ Departamento de Reumatología Centro Médico Nacional 20 de Noviembre, Instituto de Seguridad y Servicios Sociales de los Trabajadores del Estado (ISSSTE), Ciudad de México, Mexico; ^7^ Centro Universitario de Ciencias de la Salud, Departamento de Fisiología, Universidad de Guadalajara, Guadalajara, Mexico; ^8^ Centro Universitario de Ciencias de la Salud, Departamento de Biología Molecular y Genómica, Universidad de Guadalajara, Guadalajara, Mexico

**Keywords:** idiopathic inflammatory myopathies (IIM), autoantibodies, prevalence, latitude, UV radiation

## Abstract

The idiopathic inflammatory myopathies (IIM) are characterized by muscular weakness, cutaneous manifestations, muscle damage revealed by increase of muscular enzymes, muscle biopsy, electromyography and changes on magnetic resonance imaging. However, the hallmark of these IIM, is the development of myositis specific antibodies (MSA) or myositis associated antibodies (MAA). The theories about their presence in the serum of IIM is not known. Some studies have suggested that some of these MSA, such as anti-Mi-2 increases according to the intensity of UV radiation. There is scarce information about the environmental factors that might contribute in order to be considered as triggering factors as UV radiation might be. In this review, we analyzed the reported prevalence of MSAs and MAAs regarding to their geographical location and the possible relation with UV radiation. We collected the prevalence data of fifteen MSA and thirteen MAA from 22 countries around the world and we were able to observe a difference in prevalence between countries and continents. We found differences in anti-PL7, anti-Ro52, anti-La and anti-Ku prevalence according to UV radiation level. Otherwise, we observed that anti-Mi-2 prevalence increases near to the Equator meanwhile anti-MJ/NXP2 and anti-ARS prevalence had an opposite behavior increasing their prevalence in the geographical locations farther to the Equator. Our results highlighted the importance to include the UV radiation and other environmental factors in IIM studies, in order to clarify its association with MSA and MAA prevalence as well as its possible role in the immunopathogenesis of these diseases.

## Introduction

The idiopathic inflammatory myopathies (IIM), also known as myositis, represent a heterogeneous group of autoimmune rheumatic diseases. The main features are muscle weakness, multiorgan involvement including skin, joints, lungs, heart and gastrointestinal tract, as well as malignancy development (1, 2).

One of the more recent classification criteria of myositis, is the Classification Criteria for Adult and Juvenile Idiopathic Inflammatory Myopathies established by the European League Against Rheumatism/American College of Rheumatology (EULAR/ACR) in 2017. According to this, the myositis subgroups encompass dermatomyositis (DM), amyopathic dermatomyositis (ADM), juvenile dermatomyositis (JDM), polymyositis (PM), inclusion body myositis (IBM), immune-mediated necrotizing myopathy (IMNM) and juvenile myositis (JM) (3).

**Figure 1:**
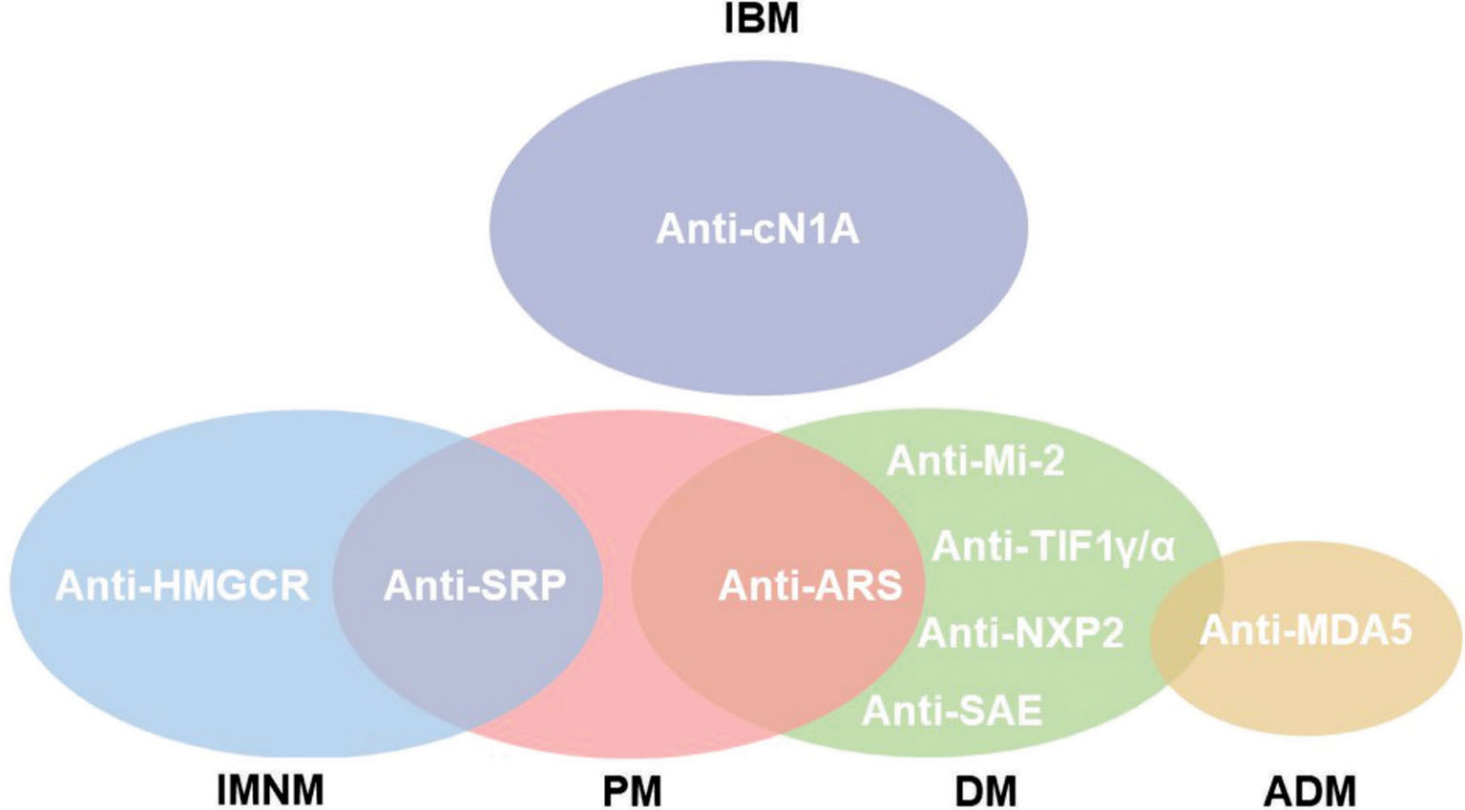
Subgroups of IIM according to autoantibody phenotype. Not all autoantibodies are exclusive for the myopathy subgroup, as is the case of anti-Signal Recognition Particle (SRP) that might be found in polymyositis (PM) and immune-mediated necrotizing myopathy (IMNM). Notwithstanding, anti-hydroxymethylglutaryl coenzyme A reductase (HMGCR) is classically observed in IMNM. The anti-aminoacyl tRNA synthetase (ARS) autoantibodies are related to anti-synthetase syndrome (ASSD). Inclusion body myositis (IBM) is associated but not exclusive for anti-cytosolic 5’nucleotidase 1A (cN1A). Dermatomyositis (DM) is associated to cancer development in positive patients for anti-Transcription Intermediary Factor 1γ/α (TIF1γ/α). Anti-Mi-2, Nuclear Matrix Protein 2 (NXP2) and anti-Small ubiquitin-like modifier Activating Enzyme (SAE) are also related to DM. The presence of anti-Melanoma Differentiation-Associate Gene 5 (MDA5) is associated with rapidly progressive interstitial lung disease (RPILD) in amyopathic dermatomyositis (ADM).

These subgroups differ in age, clinical manifestations and histopathological features. IIM are associated with the presence of myositis specific antibodies (MSA) and myositis associated antibodies (MAA) (**Figure 1**) (4).

Environment and genetics might be involved in myositis pathogenesis. UV radiation has been identified as a risk factor for DM development (5). The geographic distribution of MSA for Mi-2 and its association with UV radiation and proximity to the equator area is observed (6–9).

### UV as a Triggering Factor for Autoimmunity in Dermatomyositis

UV light is classified according to wavelength into UVA (315 – 400 nm), UVB (280 – 315 nm) and UVC (200 – 280 nm). In the case of UVB, it does not penetrate deeper than epidermis and is strongly absorbed by DNA and proteins, therefore it is an inducer of DNA damage of keratinocyte resulting in apoptosis and antigen re-localization (10, 11).

Other important aspect of UVB radiation, is that promotes the expression of adhesion molecules as well as keratinocyte stimulation to produce IL-1, IL-6, IL-8, IL-10, GM-CSF, TNF-α and IFN-I (12, 13). These cytokines might influence the type I IFN signature in IIM through the myocytes overexpression of MHC class I molecules as well as B cell activating factor belonging to the TNF family (BAFF) activation (14).

#### Prevalence of Anti-Mi-2, UV Radiation and Geographical Distribution

Anti-Mi-2 is an autoantibody mostly present in DM. Mi-2 antigen has two recognized isoforms named as alpha and beta; encoded by the chromodomain helicase DNA binding protein 3 (*CHD3*) and 4 (*CHD4*) genes, respectively. Mi-2 is part of the nucleosome and remodeling deacetylase (NuRD) complex, which is characterized by its different enzymatic activities: ATP-dependent nucleosome remodeling and histone deacetylase activity (15). Mi-2 as part of the complex NuRD, has been reported to show a check-point like activity during DNA replication as important factor for the stability of pericentric heterochromatin (16).

Using cell lines of male and female keratinocytes, it was demonstrated that UVB radiation increases the Mi-2 antigen expression (17). Notwithstanding, other studies have demonstrated that MSA autoantibodies are not dependent of the level of gene transcription for their particular autoantigen (18). In the last years, collaborative worldwide groups have been published studies dealing with clinical characteristics according to IIM classification.

Since it has been reported that Mexico has the highest prevalence of anti-Mi-2 autoantibodies (9, 19). In this review paper, we focused on literature regarding prevalence of MSA and MAA according to geographical location.

## Methods

For the references revision, we used the PubMed and medical subject headings (MeSH) data base from the National Center for Biotechnology Information (NCBI). We considered the publication dates from 10/01/1999 to 10/07/2019 (8). With regard to search criteria, we included clinical studies, meta-analysis, observational studies and twin studies published only in English language.

**Figure 2:**
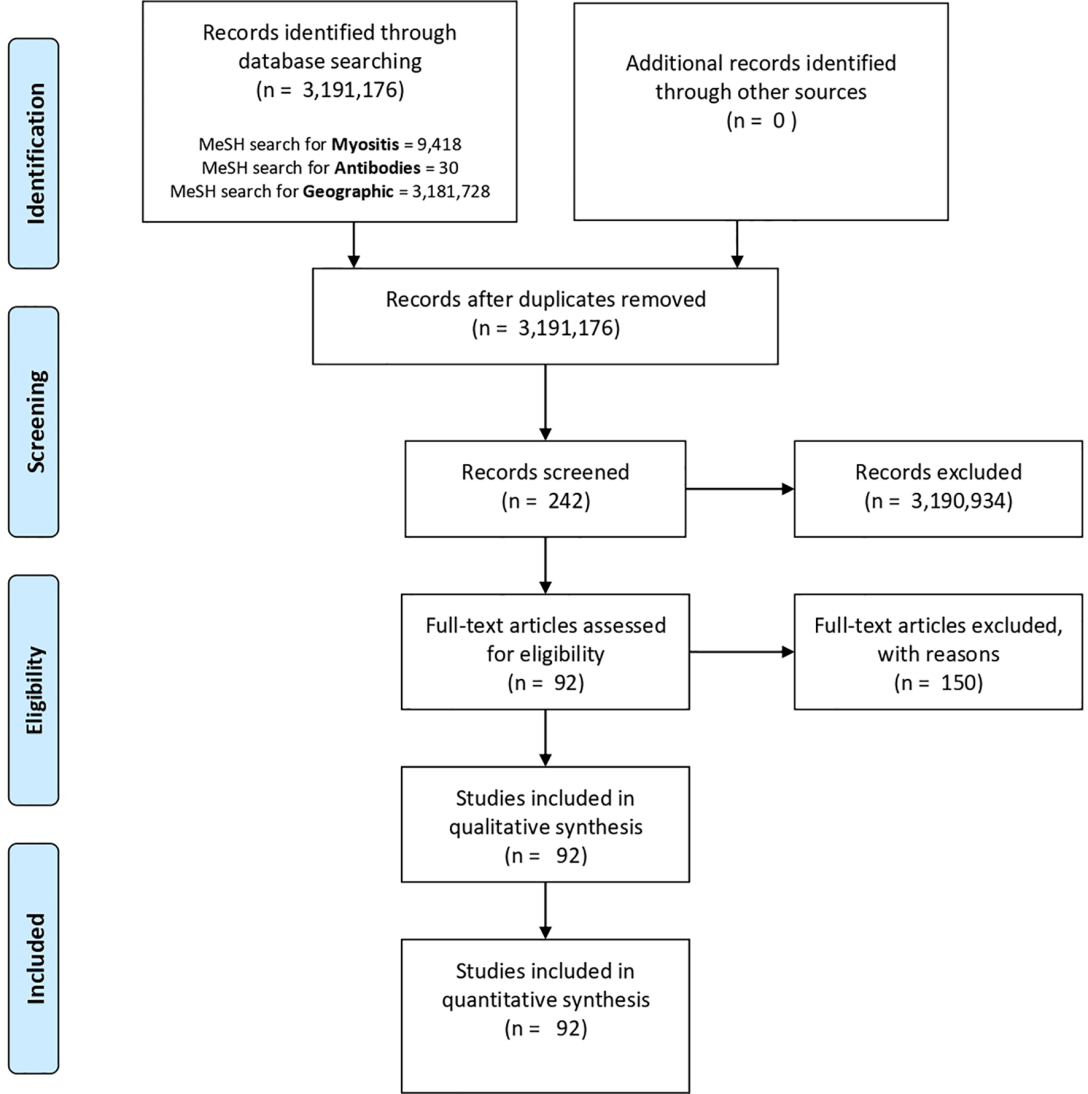
PubMed and MeSH database PRISMA flow diagram. Three key aspects were considered in our database search: myositis (myositis, polymyositis, inclusion body myositis or dermatomyositis), geographical location (epidemiology or geographic location) and antibodies (antibodies or anti-Mi-2). However, the only screened records were those papers related to the three important aspects in our search (n = 242). Finally, 150 of them were excluded and 92 were considered and reviewed in this study.

We considered three key aspects: myositis, geographical location and antibodies in the MeSH database (**Figure 2**). We did the PubMed search on October 8th, at that moment, the final algorithm for our search in PubMed was:

(((“Myositis”[Mesh] OR “Myositis, Inclusion Body”[Mesh] OR “Polymyositis”[Mesh] OR “Dermatomyositis”[Mesh]) AND (“1999/10/01”[PDAT]: “2019/10/07”[PDAT])) AND ((“Geographic Locations”[Mesh] OR “epidemiology”[Subheading]) AND (“1999/10/01”[PDAT]: “2019/10/07”[PDAT]))) AND ((“Mi-2 antibodies”[Supplementary Concept] OR “Antibodies”[Mesh]) AND (“1999/10/01”[PDAT]: “2019/10/07”[PDAT])) AND ((“1999/10/01”[PDAT]: “2019/10/07”[PDAT]) AND English[lang])

As a result of the final search, we obtained 242 articles. However, not all of them were included for this review (**Supplementary Table 1**). The exclusion criteria were: lack of relation with the central topic, absence or unclear prevalence of MSAs and MAAs, as well as overlapping with other diseases. In the case of multicentric studies they were considered only if the number of subjects per city was more than 10, including the autoantibody prevalence. Meta-analysis and review papers, were excluded because of data duplication. The final number of considered articles for this review was 92 (6, 9, 20–108).

We obtained the monthly city UV average from the website: https://www.worldweatheronline.com/. Initially, we tried to obtain the annual UV average of the year of the publication date or the year when the study was carried out; however, in some articles the period of collection data was very wide. The considered time for our reference search was established from 1999 to 2019, and we decided to consult the 2010 monthly UV average. From these data, we calculated the measures of central tendency including mean and median as well as the minimal and maximal UV radiation per year of every city.

Once we obtained the UV radiation level data, we classified them according to a collaboration of: World Health Organization, World Meteorological Organization, United Nations Environment Program and the International Commission on Non-Ionizing Radiation Protection. Then the Global Solar UV Index (UVI) that describes the level of solar UV radiation at Earth’s surface and its values were grouped into exposure categories in the next ranges: a) low: < 2; b) moderate: 3 to 5; c) high: 6 to 7; d) very high: 8 to 10 and; e) extreme: up to 11.

We also consider the latitude of every city from the website: (https://www.geodatos.net/en), afterwards, we classified the prevalence according to geographical areas: a) Antarctic polar circle to Tropic of Capricorn; b) Tropic of Capricorn to Equator; c) Equator to Tropic of Cancer, and; d) Tropic of Cancer to Artic polar circle.

All the autoantibody prevalence from the reviewed articles as well as the UV and latitude values, were analyzed by SPSS v.22 and graphed as appropriate by GraphPad Prism v.6 software. The obtained data was reported in tables as mean, minimum and maximum. The non-parametric (Kolmogorov-Smirnov, Mann-Whitney U, Kruskal-Wallis, Exact Fisher and Spearman’s Rho) statistics tests were carried out due to the type of variables and sample size. A *P* value lower than 0.05 was considered significant.

## Results

### The Prevalence of MSA but Not MAA Is Distinct According to IIM Phenotype

Within the 92 selected articles, we firstly identified the MSA and MAA prevalence, being statistically different for anti-Mi-2, Anti-MDA5/CADM140, anti-MJ/NXP2, Anti-TIF1α/γ, anti-SAE and anti-SRP. The IIM subgroups included JDM, JPM, DM, PM, IBM and IMNM (**Tables 1** and **2**).

**Table 1:** Global MSA prevalence according to IIM subgroup.

MSA $\bar{x}$ (min – max)	IIM SUBGROUP							*P* ^#&^
	JDM (n = 6)	JPM (n = 2)	DM (n = 31)	ADM (n = 5)	PM (n = 14)	IBM (n = 5)	IMNM (n = 4)	
**Anti-ARS**	8.2 (2.8 – 13.6) IP-DID, LB	9.1 IP-DID	18.5 (7.4 – 46.4) IP, IPB, LB	0.0 IPB	28.7 (12.0 – 53.8) IP, IPB, LB	---	---	0.176*
**Anti Jo-1**	6.7 (3.0 – 25.8) ID, IP-DID, LB	9.1 IP-DID	11.3 (0.0 – 33.3) ELISA, IP, IPB, IP-DID, LB.	0.0 (0.0 – 0.0) LB, IPB	16.7 (2.0 – 25.8) IP, IPB, IP-DID, LB, MR	0.0 MR	---	0.090*
**Anti-PL7**	0.5 (0.0 – 1.1) ID, IP	---	2.8 (0.8 – 4.9) IP, LB	0.0 NR	0.8	1.8 ELISA	---	0.500**
**Anti-PL12**	0.4 (0.0 – 0.8) ID, IP-DID	0.0 IP-DID	1.7 (0.0 – 5.4) IP, LB	0.0 NR	IP 2.5 (0.0 – 5.0)	---	---	0.786*
**Anti-EJ**	0.1 (0.0 – 0.3) ID, IP-DID	0.0 IP-DID	2.2 (0.0 – 5.9) IP, LB	---	IP 2.8 (0.0 – 5.0)	---	---	0.545*
**Anti-OJ**	---	---	0.4 (0.0 – 0.9) IP	---	IP 3.7 (0.8 – 6.7)	---	---	0.500**
**Anti-KS**	0.3 IP-DID	0.0 IP-DID	0.9 IP	---	IP 0.8 IP	---	---	---
**Anti-Mi-2**	5.2 (2.8 – 10.0) ID, IP, IP-DID, LB	0.0 (0.0 – 0.0) IP-DID, IP	16.5 (2.4 – 59.0) IPB, IP, LB	0.0 (0.0 – 0.0) LB, IPB	1.5 (0.0 – 8.0) IPB, IP, LB	---	---	<0.001*
**Anti-MDA5/CADM140**	9.4 (6.7 – 12.2) ID, IP-ELISA	---	13.6 (0.0 – 45.6) ELISA, ELISA-IPB, ELISA-ID, IP, IPB, IP-ID-ELISA, LB, NR	48.5 (27.3 – 67.0) ELISA, ELISA-ID, IP, IPB	0.0 (0.0 – 0.0) ELISA, ELISA-IPB, IP, IPB, IP-ID-ELISA, LB	---	---	<0.001*
**Anti-MJ/NXP2**	23.6 (16.7 – 32.5) IP-ELISA, IP-ELISA-IPB, IP-IPB	18.8 (9.1 – 28.6) IP-DID-IPB, IP-IPB	13.8 (4.0 – 23.8) IPB, IP	---	0.5 (0.0 – 1.9) IPB, IP	---	---	0.002**
**Anti-TIF1α/γ (p155/140)**	21.3 (3.0 – 34.7) ID, IP, IP-DID-IPB	0.0 (0.0 – 0.0) IP-DID-IPB, IP	22.5 (6.6 – 39.2) IPB, IP	36.4 NR	0.7 (0.0 – 3.3) IPB, IP	---	---	< 0.001**
**Anti-HMGCR**	---	---	0.0 (0.0 – 0.0) ELISA-IP, IP	---	0.0 (0.0 – 0.0) ELISA-IP, IP	4.2 ELISA-IP	38.0 (30.8 – 45.2) ELISA-IP, LBI	0.200**
**Anti-SAE**	0.7 (0.3 – 1.1) IP, IP-DID	0.0 IP-DID	2.0 (0.0 – 4.0) IPB, IP	---	0.0 (0.0 – 0.0) IPB, IP	---	---	0.033**
**Anti-SRP**	4.2 (0.0 – 10.0) ID, IP, IP-DID, LB	18.2 IP-DID	2.1 (0.0 – 6.0) IP, IPB, LB	0.0 (0.0 – 0.0) IPB, NR	8.4 (3.8 – 21.1) IP, IPB, LB	0.0 LB	26.3 (0.0 – 42.6) LBI, LB	0.019*
**Anti-cN1A**	---	---	---	---	---	53.4 (34.8 – 72.0) ELISA, ELISA-IPB	---	---

Total number of reports = 67; $\bar{x}$, mean; min, minimum; max, maximum; n, number of reports by subgroups; MSA, Myositis Specific Autoantibodies; IIM, Idiopathic Inflammatory Myopathies; JDM, Juvenile Dermatomyositis; JPM, Juvenile Polymyositis; DM, Dermatomyositis; PM, Polymyositis; IBM, Inclusion Body Myositis; IMNM, Immune-Mediated Necrotizing Myopathy; ARS, Aminoacyl tRNA Synthetase; MDA5, Melanoma Differentiation-Associated gene 5; NXP, Nuclear Matrix Protein; TIF1γ/α, Transcription Intermediary Factor 1γ/α,; HMGCR, Hydroxymethylglutaryl coenzyme A reductase; SAE, Small ubiquitin-like modifier Activating Enzyme; SRP, Signal Recognition Particle; cN1A, cytosolic 5’ nucleotidase 1A; IP, Immunoprecipitation; DID, Ouchterlony Double Immunodifussion; LB, Line-blot; ID, Immunodot; MR, Medical Records; ELISA, Enzyme-linked Immunosorbent Assay; IPB, Immunoprecipitation-blotting; NR, No reported; LBI, Laser Bead Immunoassay. ^#^Only IIM subgroups with n ≥ 2 were compared; ^&^Non-parametric tests as appropriate; *Asymptotic; **Exact Fisher test. Bold values are for highlighting the P values lower than 0.05 (significative).

**Table 2:** Global MAA prevalence according to IIM subgroup.

MAA $\bar{x}$ (min – max)	IIM SUBGROUP							*P* ^#&^
	JDM (n = 6)	JPM (n = 2)	DM (n = 31)	ADM (n = 5)	PM (n = 14)	IBM (n = 5)	IMNM (n = 4)	
**Anti-RNP**	---	---	---	---	---	---	---	---
- **Anti-U1RNP**	3.9 (2.0 – 7.5) IP, IP-DID	6.0 (0.0 – 12.1) IP-DID, IP	4.2 (0.0 – 7.4) IP, IPB, IP-DID	0.0 IPB	4.3 (2.9 – 6.7) IP, IPB, IP-DID	---	---	0.988**
- **Anti-U3RNP**	---	---	1.9 IP	0.0 NR	0.0 IP	---	---	---
- **Anti-U5RNP**	---	---	---	---	---	---	---	---
**Anti-Ro**	2.7 (0.0 – 5.4) ID, IP-DID	6.1 IP-DID	5.7 (0.4 – 11.1) IPB, IP	13.5 IPB	4.9 IPB	---	---	0.667**
- **Anti-Ro52**	22.7 LB	---	19.0 (0.0 – 29.0) ELISA, LB	---	22.0 (0.0 – 44.0) LB	5.3 (0.0 – 10.7) ELISA, LB	11.2 (8.7 – 13.7) LB	0.641**
- **Anti-Ro60**	---	---	13.8 (6.5 – 24.0) IP	---	---	---	---	---
**Anti-La**	1.5 (0.0 – 3.0) ID, IP-DID	0.0 IP-DID	---	---	---	---	---	---
**Anti-PMScl**	3.8 (2.5 – 6.7) ID, IP, IP-DID	3.0 (0.0 – 6.1) IP-DID	6.9 (0.0 – 13.1) IP, IP-DID	0.0 LB	4.8 (0.0 – 10.0) IP, IP-DID	---	---	0.817**
- **Anti-PMScl75**	13.6 LB	---	6.3 (0.0 – 12.7) LB	---	8.0 (0.0 – 16.0) LB	0.0 LB	12.0 (2.3 – 21.7) LB	0.733**
- **Anti-PMScl100**	4.5 LB	---	1.8 LB	---	4.0 LB	1.8 ELISA	---	---
**Anti-Ku**	3.5 (0.0 – 13.6) ID, IP-DID, LB	0.0 (0.0 – 0.0) IP-DID, IP	3.0 (0.0 – 12.7) IP, LB	0.0 NR	3.1 (0.0 – 8.8) IP, LB	---	4.3 LB	0.451**
**Anti-Su**	---	---	---	---	---	---	---	---

Total number of reports = 28; $\bar{x}$, mean; min, minimum; max, maximum; n, number of reports by subgroups; MAA, Myositis Associated Autoantibodies; IIM, Idiopathic Inflammatory Myopathies; JDM, Juvenile Dermatomyositis; JPM, Juvenile Polymyositis; DM, Dermatomyositis; PM, Polymyositis; IBM, Inclusion Body Myositis; IMNM, Immune-Mediated Necrotizing Myopathy; RNP, ribonucleoprotein; IP, Immunoprecipitation; DID, Ouchterlony Double Immunodifussion; ID, Immunodot; LB, Line-Blot; ELISA, Enzyme-linked Immunosorbant Assay; NR, No reported. ^#^Only IIM subgroups with n ≥ 5 were compared; ^&^Non-parametric tests as appropriate; **Exact Fisher test.

### Not All the MSA Differ on Prevalence Between Countries, Continents or Geographical Areas

We obtained information of MSA and MAA prevalence from 22 countries around the world (**Tables 3–5**). Interestingly, when we compared the prevalence between countries, we only find differences in some autoantibodies: anti-SRP between European countries (*P* = 0.043) (**Table 3**), anti-PL12 (*P* = 0.036) in Asia (**Table 4**) and anti-MJ/NXP2 in American continent (*P* = 0.003) (**Table 5**). However, when we grouped and compared the prevalence between continents, we found differences in some MSA such as anti-aminoacyl tRNA synthetases (ARS) (*P* = 0.015), anti-Jo-1 (*P* = 0.049), anti-PL7 (*P* = 0.017) and anti-MJ/NXP2 (*P* = 0.023) (**Supplementary Table 2**).

**Table 3:** MSA and MAA prevalences in Europe and Oceania.

MSA/MAA $\bar{x}$ (min – max)	EUROPE AND OCEANIA												*P^#&^*
	UK (n = 7)	SW (n = 2)	CZ (n = 3)	NT (n = 1)	GM (n = 1)	FR (n = 4)	PL (n = 1)	HG (n = 5)	IT (n = 8)	SP (n = 5)	GR (n = 1)	AS (n = 4)	
**Anti-ARS**	13.3 DID-IIP-IP	---	---	---	40.1 DID-IIF-IP	---	3.7 DID-IIF-IP	---	10.6 DID-IIF-IP	20.6 (18.7 – 23.9) ELISA, IP, IB/ELISA	---	3.4 LB	---
- **Anti Jo-1**	20.3 (18.6 – 22.2) DB-IIF-ELISA, IP, IP-DID, MR	14.8 (0.0 – 33.3) LB, MR	18.5 (0.0 – 27.4) LB	20.0 IPB	---	13.9 (7.1 – 26.3) DID-ELISA, MR	---	17.0 (13.8 – 18.5) ELISA-LB, LB, LB-IP, NR	9.6 (0.0 – 24.5) IP, ID, IPB, LB-IPB, NR	7.9 (0.0 – 15.9) ELISA, LB NR	22.1 LB	4.7 (1.7 – 7.8) LB, MR	0.307*
- **Anti-PL7**	0.6 (0.0 – 1.1) IP	---	---	---	---	2.7 MR	---	1.3 (1.2 – 1.5) LB, LB-IP	3.4 IPB	0.0 NR	1.0 LB	2.2 (1.8 – 2.6) ELISA, MR	0.200**
- **Anti-PL12**	0.4 (0.0 – 0.9) IP	---	---	---	---	1.4 MR	---	0.3 (0.3 – 0.3) LB, LB-IP	3.4 (0.0 – 5.1) IP, IPB	0.0	3.2 LB	1.7 (1.7 – 1.7) LB, MR	0.733**
- **Anti-EJ**	0.7 (0.0 – 1.1) IP, MR	--	---	---	---	---	---	---	3.5 (0.0 – 5.9) IP	---	1.0 LB	---	0.161**
- **Anti-OJ**	0.8 (0.8 – 0.9) IP	--	---	---	---	---	---	---	2.3 (0.0 – 6.7) IP, IPB	---	1.0 LB	---	0.467**
- **Anti-KS**	0.8 (0.8 – 0.9) IP	--	---	---	---	---	---	---	---	---	---	---	---
**Anti-Mi-2**	6.4 (0.8 – 15.7) DID-IIF-IP, IP, MR	---	6.7 LB-WB	6.0 ELISA	13.6 DID-IIF-IP	---	3.7 DID-IIF-IP	7.7 (7.7 – 7.7) LB, LB-IP, NR	6.1 (0.0 – 19.1) IP, DID-IIF-IP, ID, LB-IPB	3.4 (0.0 – 6.8) IP, LB	---	3.0 (2.5 – 3.5) LB, MR	0.452**
**Anti-MDA5/CADM140**	12.2 IP-ELISA	---	---	---	---	---	---	---	3.7 (0.0 – 14.7) ELISA-IP-IPB, IP	28.8 (5.7 – 53.3) ELISA-IB, IP	1.0 LB	---	0.086**
**Anti-MJ/NXP2**	16.7 IP-ELISA	---	---	---	---	---	---	1.2 (1.2 – 1.2) IP	11.5 (0.0 – 23.5) IP, IP-ELISA-IPB	---	4.2 LB	---	0.714**
**Anti-TIF1α/γ (p155/140)**	20.0 IP	---	8.2 IP	---	---	---	---	3.6 (3.6 – 3.6) IP	6.5 (0.0 – 22.2) IP, ID	36.4 NR	7.4 LB	---	0.810**
**Anti-HMGCR**	---	---	6.9 ELISA	---	---	45.2 LBI	---	---	0.0 (0.0 – 0.0) IP	---	---	---	---
**Anti-SAE**	2.6 (1.1 – 4.1) IP	---	---	---	---	---	---	1.2 (1.2 – 1.2) IP	5.8 (4.2 – 7.5) ID, IPB	---	---	---	0.200**
**Anti-SRP**	2.3 (0.0 – 4.3) DID-IIF-IP, IP, MR	---	1.9 LB	4.0 IP	0.0 DID-IIF-IP	22.6 (2.7 – 42.6) LBI, MR	0.0 DID-IIF-IP	4.2 (4.2 – 4.2) LB-IP, LB	5.3 (2.1 – 10.0) IP, DID-IIF-IP, IPB	0.0 (0.0 – 0.0) IP, NR	12.6 LB	1.2 (0.0 – 2.5) LB	0.043**
**Anti- cN1A**	---	---	---	---	---	---	---	---	---	---	---	34.8 ELISA	---
**Anti-RNP**	---	---	---	---	---	---	---	---	0.6 NR	12.4 ELISA/CI	---	---	---
- **Anti-U1RNP**	4.6 (2.2 – 7.4) IP, IP-DID	---	---	---	---	---	---	2.3 LB	5.3 (3.4 – 6.7) IP, IPB	6.8 ELISA	---	---	> 0.999**
- **Anti-U3RNP**	0.9 (0.0 – 1.9) IP	---	---	---	---	---	---	---	---	0.0 LB	---	---	---
- **Anti-U5RNP**	---	---	---	---	---	---	---	---	---	2.3 IP	---	---	---
**Anti-Ro**	---	---	11.5 LB-WB	---	---	---	---	8.5 ELISA	5.2 (3.4 – 8.5) IP, IPB, NR	12.1 ELISA/CI	---	---	---
- **Anti-Ro52**	---	---	16.6 (0.5 – 32.7) LB, LB-WB	---	---	---	---	---	23.6 LB-IPB	20.4 IP	29.5 LB	15.0 (8.7 – 29.6) ELISA, LB, MR	---
- **Anti-Ro60**	---	---	---	---	---	---	---	---	---	22.7 IP	---	---	---
**Anti-La**	---	---	---	---	---	---	---	5.4 ELISA	1.4 (1.2 – 1.7) IPB, NR	---	---	---	---
**Anti-PMScl**	5.5 (4.3 – 6.7) IP, IP-DID	---	12.3 LB-WB	---	---	---	---	---	8.3 (4.3 – 10.3) IP, LB-IPB	14.2 (0.0 – 31.2) IP, LB	---	8.7 MR	0.524**
- **Anti-PMScl75**	---	---	---	---	---	---	---	---	---	---	9.5 LB	21.7 LB	---
- **Anti-PMScl100**	---	---	---	---	---	---	---	---	---	---	4.2 LB	1.8 ELISA	---
**Anti-Ku**	0.9 (0.0 – 1.9) IP	--	0.5 LB	---	---	---	---	3.8 LB	4.8 LB-IPB	0.5 (0.0 – 1.1) NR	6.3 LB	3.4 (2.6 – 4.3) LB, MR	> 0.999**
**Anti-Su**	---	--	---	---	---	---	---	---	3.4 IP	---	---	---	---

$\bar{x}$, mean; min, minimum; max, maximum; n, number of articles per country; MSA, Myositis Specific Autoantibodies; IIM, Idiopathic Inflammatory Myopathies; DM, Dermatomyositis; PM, Polymyositis; IBM, Inclusion Body Myositis; IMNM, Immune-Mediated Necrotizing Myopathy; ARS, Aminoacyl tRNA Synthetase; MDA5, Melanoma Differentiation-Associated Gene 5; NXP, Nuclear Matrix Protein; TIF1γ/α, Transcription Intermediary Factor 1γ/α; HMGCR, Hydroxymethylglutaryl coenzyme A reductase; SAE, Small ubiquitin-like modifier Activating Enzyme; SRP, Signal Recognition Particle; cN1A, cytosolic 5’ nucleotidase 1A; RNP, ribonucleoprotein; UK, United Kingdom; SW, Sweden; CZ, Czech Republic; NT, Netherlands; GM, Germany; FR, France; PL, Poland; HG, Hungary; IT, Italy; SP, Spain; GR, Greece; AS, Australia; DID, Ouchterlony Double Immunodifussion; IIF, Indirect Immunofluorescence; IP, Immunoprecipitation: DB, Dotblot; ELISA, Enzyme-Linked Immunosorbant Assay; FEI, Fluorescence Enzyme Immunoassay; MR, Medical Records; IPB, Immunoprecipitation-blotting; NR, No Reported; WB, Western Blot; LBI, Laser Bead Immunoassay; IB, Immunoblot; CI, Chemiluminiscence Immunoassay. ^&^Non-parametric tests as appropriate; *Asymptotic; **Exact Fisher test. ^#^Only countries from Europe were compared. Bold values are for highlighting the P values lower than 0.05 (significative).

**Table 4:** MSA and MAA prevalences in Asia.

MSA/MAA $\bar{x}$ (min – max)	ASIA				*P^&^*
	SK (n = 2)	JP (n = 27)	CH (n = 11)	IN (n = 2)	
**Anti-ARS**	13.9 (12.2 – 15.7) DID-IIF-IP, IP	27.1 (0.0 – 54.1) ELISA, IP, ELISA-IP, IPB	24.8 (14.0 – 53.8) IP, LB	12.8 (12.0 – 13.6) LB	0.120*
- **Anti Jo-1**	---	17.6 (0.0 – 48.5) DID-IP, ELISA, FEI, ELISA-IP, IP, IPB, MR	12.5 (7.0 – 19.2) LB	13.8 (3.0 – 24.0) ID, LB	0.588**
- **Anti-PL7**	---	11.3 (3.6 – 16.7) ELISA-IP, IP	3.7 (1.4 – 4.9) LB	0.0 ID	0.083**
- **Anti-PL12**	---	6.2 (2.0 – 9.1) ELISA-IP, IP	1.2 (0.4 – 1.9) LB	0.0 ID	0.036**
- **Anti-EJ**	---	11.3 (3.0 – 30.3) ELISA-IP, IP	2.2 (1.4 – 3.0) LB	0.0 ID	0.086**
- **Anti-OJ**	---	1.5 (0.0 – 3.0) IP	---	---	---
- **Anti-KS**	---	8.1 (6.2 – 10.0) ELISA-IP, IP	---	---	---
**Anti-Mi-2**	11.0 (7.8 – 14.3) DID-IIF-IP, IP	3.2 (0.0 – 10.9) IPB, IP, WB	3.7 (0.0 – 7.4) LB	13.5 (3.3 – 38.2) ID, LB	0.068**
**Anti-MDA5/CADM140**	---	19.6 (0.0 – 67.0) ELISA, IP, IPB, IP-ELISA, IP-IB-ELISA	14.6 (0.0 – 45.6) ELISA, LB, LB-ELISA	6.7 ID	0.632**
**Anti-MJ/NXP2**	---	7.0 (0.0 – 16.0) IPB, WB	5.8 (1.9 – 8.8) IPB, IP, LB	---	0.886**
**Anti-TIF1α/γ (p155/140)**	17.3 IP	12.1 (0.0 – 21.7) ELISA, IPB, WB, IP	21.5 (0.0 – 52.8) IPB, LB	3.0 ID	> 0.999**
**Anti-HMGCR**	---	7.8 (0.0 – 30.8) ELISA-IP, NR	14.0 (6.6 – 21.4) ELISA, IP	---	0.333**
**Anti-SAE**	---	1.1 (0.0 – 1.8) IPB	2.0 (0.0 – 5.5) IPB, IP, LB	---	0.893**
**Anti-SRP**	3.9 (2.0 – 5.9) DID-IIF-IP, IP	5.7 (0.0 – 21.1) IP, IPB	7.2 (0.0 – 36.4) IP, LB	6.0 (4.0 – 10.0) ID, LB	0.653**
**Anti- cN1A**	---	---	---	---	---
**Anti-RNP**	---	---	0.8 LB	---	---
- **Anti-U1RNP**	---	5.2 (0.0 – 14.9) DID, IPB, IP	---	---	---
- **Anti-U3RNP**	---	---	---	---	---
- **Anti-U5RNP**	---	---	---	---	---
**Anti-Ro**	---	13.5 (0.4 – 44.8) DID-IP, IP, IPB	16.8 (16.2 – 17.5) LB	0.0 ID	0.286**
- **Anti-Ro52**	---	46.4 ELISA	16.4 (0.0 – 50.8) LB	30.7 (22.7 – 44.0) LB	0.375**
- **Anti-Ro60**	---	---	6.0 LB	---	---
**Anti-La**	---	1.6 DID-IP	1.9 (1.7 – 2.2) LB	3.0 ID	---
**Anti-PMScl**	---	0.0 (0.0 – 0.0) IP	---	3.3 ID	---
- **Anti-PMScl75**	---	3.9 ELISA-IP	0.6 (0.0 – 2.3) LB	14.1 (12.7 – 16.0) LB	0.057**
- **Anti-PMScl100**	---	3.9 ELISA-IP	---	3.4 (1.8 – 4.5) LB	---
**Anti-Ku**	---	3.1 (0.0 – 8.8) IP	0.6 (0.0 – 1.2) IP	7.6 (0.0 – 13.6) ID, LB	0.421**
**Anti-Su**	---	5.8 IP	---	---	---

$\bar{x}$, mean; min, minimum; max, maximum; n, number of articles per country; MSA, Myositis Specific Autoantibodies; IIM, Idiopathic Inflammatory Myopathies; DM, Dermatomyositis; PM, Polymyositis; IBM, Inclusion Body Myositis; IMNM, Immune-Mediated Necrotizing Myopathy; ARS, Aminoacyl tRNA Synthetase; MDA5, Melanoma Differentiation-Associated Gene 5; NXP, Nuclear Matrix Protein; TIF1γ/α, Transcription Intermediary Factor 1γ/α; HMGCR, Hydroxymethylglutaryl coenzyme A reductase; SAE, Small ubiquitin-like modifier Activating Enzyme; SRP, Signal Recognition Particle; cN1A, cytosolic 5’ nucleotidase 1A; RNP, ribonucleoprotein; SK, South Korea; JP, Japan; CH, China; IN, India; DID, Ouchterlony Double Immunodifussion; IIF, Indirect Immunofluorescence; IP, Immunoprecipitation; DB, Dotblot; ELISA, Enzyme-Linked Immunosorbant Assay; FEI, Fluorescence Enzyme Immunoassay; MR, Medical Records; IPB, Immunoprecipitation-blotting; NR, No Reported; WB, Western Blot; LBI, Laser Bead Immunoassay; IB, Immunoblot; CI, Chemiluminiscence Immunoassay. ^&^Non-parametric tests as appropriate; *Asymptotic; **Exact Fisher test. Bold values are for highlighting the P values lower than 0.05 (significative).

**Table 5:** MSA and MAA prevalences in America.

MSA/MAA $\bar{x}$ (min – max)	AMERICA						*P^&^*
	CN (n = 1)	CE (n = 1)	USA (n = 14)	MX (n = 2)	GM (n = 1)	AG (n =2)	
**Anti-ARS**	3.2 DID-IIF-IP	23.1 DID-IIF-IP	10.3 (2.8 – 25.8) DID-IIF-IP, IP	5.5 (0.0 – 11.1) DID-IIF-IP	3.3 DID-IIF-IP	---	0.800**
- **Anti Jo-1**	---	---	8.4 (1.4 – 22.2) ELISA-LB-IP, IP, ELISA, MR	2.0 (0.0 – 4.0) IP-ELISA	---	8.0 LB	0.222*
- **Anti-PL7**	---	---	1.3 ELISA-LB-IP	---	---	0.0 LB	---
- **Anti-PL12**	---	---	0.9 (0.0 – 1.9) ELISA-LB-IP, IP	---	---	24.0 LB	---
- **Anti-EJ**	---	---	0.2 (0.0 – 0.3) ELISA-LB-IP, IP	---	---	---	---
- **Anti-OJ**	---	---	0.3 ELISA-LB-IP	---	---	---	---
- **Anti-KS**	---	---	0.2 (0.0 – 0.3) IP	---	---	---	---
**Anti-Mi-2**	3.2 DID-IIF-IP	23.1 DID-IIF-IP	7.9 (0.0 – 25.3) DID-IIF-IP, IP, IP-DID	30.1 (12-0 – 59.0) DID-IIF-IP, IP	60.0 DID-IIF-IP	10.0 (0.0 – 20.0) IP, LB	0.090**
**Anti-MDA5/CADM140**	---	---	12.1 (7.1 – 15.4) ELISA, IP, NR	---	---	---	---
**Anti-MJ/NXP2**	---	---	17.3 (9.1 – 23.8) IP, IP-DID-IPB	5.0 (4.0 – 6.0) IP	---	30.5 (28.6 – 32.5) IP-IPB	0.003**
**Anti-TIF1α/γ (p155/140)**	---	---	28.3 (0.0 – 39.2) IP, IP-DID-IPB	22.0 (9.0 – 35.0) IP	---	13.7 (0.0 – 27.5) IP	0.529**
**Anti-HMGCR**	---	---	6.0 ELISA-IP	---	---	---	---
**Anti-SAE**	---	---	0.2 (0.0 – 0.3) IP-DID	2.0 (0.0 – 4.0) IP	---	---	> 0.999**
**Anti-SRP**	3.2 DID-IIF-IP	0.0 DID-IIF-IP	5.8 (0.0 – 18-2) DID-IIF-IP, IP, IP-DID	2.2 (0.0 – 6.0) DID-IIF-IP, IP	0.0 DID-IIF-IP	12.0 LB	0.485**
**Anti- cN1A**	---	---	72.0 ELISA-WB	----	---	---	---
**Anti-RNP**	---	---	---	---	---	---	---
- **Anti-U1RNP**	---	---	5.6 (2.0 – 12.1) IP-DID	1.0 (0.0 – 2.0) IP	---	3.7 (0.0 – 7.5) IP	0.457**
- **Anti-U3RNP**	---	---	---	---	---	---	---
- **Anti-U5RNP**	---	---	---	---	---	---	---
**Anti-Ro**	---	---	5.6 (5.4 – 6.1) IP-DID	---	---	---	---
- **Anti-Ro52**	---	---	19.5 ELISA	25.0 (21.0 – 29.0) IP-ELISA	---	----	**---**
- **Anti-Ro60**	---	---	6.5 IP	17.5 (11.0 – 24.0) IP	---	---	**---**
**Anti-La**	---	---	0.0 (0.0 – 0.0) IP	---	---	---	
**Anti-PMScl**	---	---	6.1 (2.6 – 13.1) IP, IP-DID	7.0 (2.0 – 12.0) IP	---	1.2 (0.0 – 2.5) IP	0.248**
- **Anti-PMScl75**	---	---	---	---	---	---	---
- **Anti-PMScl100**	---	---	---	---	---	---	---
**Anti-Ku**	---	---	0.2 (0.0 – 0.3) IP-DID	---	---	0.0 (0.0 – 0.0) IP	0.300**
**Anti-Su**	---	---	---	---	---	---	---

$\bar{x}$, mean; min, minimum; max, maximum; n, number of articles per country; MSA, Myositis Specific Autoantibodies; IIM, Idiopathic Inflammatory Myopathies; DM, Dermatomyositis; PM, Polymyositis; IBM, Inclusion Body Myositis; IMNM, Immune-Mediated Necrotizing Myopathy; ARS, Aminoacyl tRNA Synthetase; MDA5, Melanoma Differentiation-Associated Gene 5; NXP, Nuclear Matrix Protein; TIF1γ/α, Transcription Intermediary Factor 1γ/α; HMGCR, Hydroxymethylglutaryl coenzyme A reductase; SAE, Small ubiquitin-like modifier Activating Enzyme; SRP, Signal Recognition Particle; cN1A, cytosolic 5’ nucleotidase 1A; RNP, ribonucleoprotein; CN, Canada; CE, Chile; USA, United States of America; MX, Mexico; GM, Guatemala; AG, Argentina; DID, Ouchterlony Double Immunodifussion; IIF, Indirect Immunofluorescence; IP, Immunoprecipitation; DB, Dotblot; ELISA, Enzyme-Linked Immunosorbant Assay; FEI, Fluorescence Enzyme Immunoassay; MR, Medical Records; IPB, Immunoprecipitation-blotting; NR, No Reported; WB, Western Blot; LBI, Laser Bead Immunoassay; IB, Immunoblot; CI, Chemiluminiscence Immunoassay. ^&^Non-parametric tests as appropriate; *Asymptotic; **Exact Fisher test. Bold values are for highlighting the P values lower than 0.05 (significative).

Considering the annual mean of UV radiation per city, we categorized all the considered countries according to UVI scale (**Supplementary Table 3**). Within the 22 countries, we found that United Kingdom and Sweden had the lowest UV average radiation while India the highest.

It is important to highlight that we obtained a mean of annual UV level per country. When we analyzed the autoantibody prevalence according to the UV, we only find differences in anti-PL7 (*P* = 0.031), anti-Ro52 (*P* = 0.013), anti-La (*P* = 0.016) and anti-Ku (*P* = 0.042) (**Table 6**). Anti-Mi-2 prevalence between UV radiation levels was not statistically significant, however, we could observe a trend to increase according to UV level (**Table 6**).

**Table 6:** MSA and MAA prevalence according to UV level.

MSA/MAA $\bar{x}$ (min – max)	UV LEVEL^$^					*P* ^&^
	2	3	4	5	6	
**Anti-ARS**	18.9 (3.2 – 40.1) DID-IIF-IP	21.3 (3.7 – 48.0) ELISA, ELISA-IP, DID-IIF-IP, IP	20.0 (0.0 – 54.1) ELISA, IP, IB/ELISA, DID-IIF-IP, IPB, LB	---	12.2 (10.6 – 13.6) DID-IIF-IP, LB	0.578*
- Anti Jo-1	17.9 (0.0 – 33.3) DB-IIF-ELISA, LB, FEI, DID-ELISA, IP, IP-IPB, IP-DID, LB-IPB, MR	11.6 (0.0 – 20.2) ELISA, ELISA-LB, IP, ELISA-IP, IPB, LB, LB-IP, LB-IPB, MR, NR	12.9 (0.0 – 48.5) DID-IP, ELISA-LB-IP, ELISA, IP, ID, LB, MR, NR	12.5 (3.0 – 22.1) ID, LB	16.3 (5.5 – 24.0) LB, MR	0.212*
- Anti-PL7	0.6 (0.0 – 1.1) IP	5.5 (1.2 – 16.7) LB, ELISA-IP, IP, IPB, LB-IP, MR	5.0 (0.0 – 16.7) ELISA, ELISA-LB,	0.5 (0.0 – 1.0) ID, LB	---	0.031**
- Anti-PL12	0.4 (0.0 – 0.9) IP	2.8 (0.0 – 8.3) ELISA-IP, IP, IPB, LB-IP, LB, MR	IP, NR, MR 3.9 (0.0 – 24.0) ELISA-LB-IP, IP, NR,	1.6 (0.0 – 3.2) ID, LB	---	0.626**
- Anti-EJ	0.7 (0.0 – 1.1) IP, MR	4.2 (0.0 – 6.2) IP, ELISA-IP	IP-DID, LB, MR 4.5 (0.0 – 30.3)	0.5 (0.0 – 1.0) ID, LB	---	0.073**
- Anti-OJ	0.8 (0.8 – 0.9) IP	2.3 (0.0 – 6.7) IP, IPB	ELISA-LB-IP, IP, IP-DID, LB	1.0 LB	---	0.839**
- Anti-KS	0.8 (0.8 – 0.9) IP	6.2 ELISA-IP	1.1 (0.0 – 3.0) ELISA-LB-IP, IP 2.6 (0.0 – 10.0) IP, IP-DID	---	---	0.467**
**Anti-Mi-2**	6.8 (0.8 – 15.7) DID-IIF-IP, ELISA, IP, LB-IPB, MR	7.0 (0.0 – 23.1) NR, IP, DID-IIF-IP, LB, LB-IPB	10.7 (0.0 – 60.0) IPB, IP, DID-IIF-IP, WB, LB, IP-DID, ID, MR	3.3^#^ ID	17.4 (4.5 – 38.2) DID-IIF-IP, LB	0.383*
**Anti-MDA5/CADM140**	20.1 (12.2 – 28.0) ELISA, IP-ELISA	14.2 (0.0 – 67.0) ELISA, ELISA-IP-IPB, IP, IP-ELISA, IP-IB-ELISA, NR	17.6 (0.0 – 53.3) ELISA, ELISA-IB, IP, IPB. IP-ELISA, LB, LB-ELISA	3.8 (1.0 – 6.7) ID, LB	15.0 (0.0 – 30.0) LB	0.723*
**Anti-MJ/NXP2**	16.3 (16.0 – 16.7) IP-IPB, IP-ELISA	9.2 (0.0 – 23.5) IP, IP-ELISA-IPB	12.8 (0.0 – 32.5) IPB, IP, WB, IP-DID-IPB, IP-IPB, LB	4.2 LB	8.3 IP	0.510**
**Anti-TIF1α/γ (p155/140)**	16.1 (8.2 – 20.0) IP-IPB, IP	10.0 (0.0 – 37.8) IP	19.1 (0.0 – 52.8) ELISA, IPB, IP, WB, NR, ID, LB, IP-DID-IPB	5.2 (3.0 – 7.4) ID, LB	---	0.344*
**Anti-HMGCR**	5.4 (4.0 – 6.9) ELISA, NR	15.1 (0.0 – 45.2) IP, LBI	10.4 (0.0 – 30.8) ELISA, ELISA-IP	---	6.6	0.903**
**Anti-SAE**	2.6 (1.1 – 4.1) IP	2.0 (1.2 – 4.2) IPB, IP	1.8 (0.0 – 7.5) IPB, IP, IP-DID-IPB, ID, LB	---	---	0.627**
**Anti-SRP**	2.5 (0.0 – 4.3) LB, IP, DID-IIF-IP, MR	6.5 (0.0 – 42.6) IP, DID-IIF-IP, IPB, LBI, LB-IP, MR	5.1 (0.0 – 36.4) IP, DID-IIF-IP, LB, IPB, IP-DID	11.3 (10.0 – 12.6) ID, LB	4.0 (2.1 – 5.5) IP	0.120*
**Anti- cN1A**	---	---	34.8 ELISA	72.0 ELISA-WB	---	---
**Anti-RNP**	---	---	4.6 (0.6 – 12.4) ELISA/CI, LB, NR	---	---	---
- **Anti-U1RNP**	4.6 (2.2 – 7.4) IP, IP-DID	4.0 (2.3 – 6.7) LB, IP, IPB	4.9 (0.0 – 14.9) ELISA, DID, IPB, IP-DID,	---	---	0.934**
- **Anti-U3RNP**	0.9 (0.0 – 1.9) IP	---	IP 0.0	---	---	---
- **Anti-U5RNP**	---	---	ELISA 2.3 ELISA-IP	---	---	---
**Anti-Ro**	11.5 LB-WB	5.2 (0.4 – 8.5) ELISA, IP, IPB	11.4 (3.7 – 44.8) DID-IP, ELISA/CI, IP, IPB, NR	0.0 ID	16.8 (16.2 – 17.5) LB	0.082**
- **Anti-Ro52**	16.6 (0.5 – 32.7) LB	29.8 (19.5 – 46.4) ELISA, LB-IPB	12.4 (0.0 – 29.6) ELISA, ELISA-IP, LB, MR	29.5 LB	37.6 (22.7 – 50.8) LB	0.013**
- **Anti-Ro60**	---	6.5 IP	15.9 (6.0 – 24.0) ELISA-IP, IP, LB	---	---	---
**Anti-La**	---	3.5 (1.7 – 5.4) ELISA, IPB	0.6 (0.0 – 1.6) DID-IP, NR	3.0 ID	1.9 (1.7 – 2.2) LB	0.016**
**Anti-PMScl**	7.2 (4.3 – 12.3) IP, IP-DID, LB-WB	5.6 (0.0 – 10.3) IP, LB-IPB	7.7 (0.0 – 31.2) IP, IP-DID, MR	3.3 ID	---	0.790**
- **Anti-PMScl75**	---	---	4.6 (0.0 – 21.7) ELISA-IP, LB	9.5 LB	14.1 (12.7 – 16.0) LB	0.167**
- **Anti-PMScl100**	---	---	2.8 (1.8 – 3.9) ELISA, ELISA-IP	4.2 LB	3.4 (1.8 – 4.5) LB	0.500**
**Anti-Ku**	0.8 (0.0 – 1.9) LB, IP	4.5 (0.5 – 8.8) LB, IP, LB-IPB	1.1 (0.0 – 4.5) IP, NR, IP-DID, LB, MR	3.1 (0.0 – 6.3) ID, LB	10.1 (4.0 – 13.6) LB	0.042*
**Anti-Su**	---	3.4 IP	5.8 IP	---	---	---

^$^The UV radiation level was calculated per city. $\bar{x}$, mean; min, minimum; max, maximum; MSA, Myositis Specific Autoantibodies; IIM, Idiopathic Inflammatory Myopathies; DM, Dermatomyositis; PM, Polymyositis; IBM, Inclusion Body Myositis; IMNM, Immune-Mediated Necrotizing Myopathy; ARS, Aminoacyl tRNA Synthetase;MDA5, Melanoma Differentiation-Associated Gene 5; NXP, Nuclear Matrix Protein; TIF1γ/α, Transcription Intermediary Factor 1γ/α; HMGCR, Hydroxymethylglutaryl coenzyme A reductase; SAE, Small ubiquitin-like modifier Activating Enzyme; SRP, Signal Recognition Particle; cN1A, cytosolic 5’ nucleotidase 1A; RNP, ribonucleoprotein; DID, Ouchterlony Double Immunodifussion; IIF, Indirect Immunofluorescence; IP, Immunoprecipitation; DB, Dotblot; ELISA, Enzyme-Linked Immunosorbant Assay; FEI, Fluorescence Enzyme Immunoassay; MR, Medical Records; IPB, Immunoprecipitation-blotting; NR, No Reported; WB, Western Blot; LBI, Laser Bead Immunoassay; IB, Immunoblot; CI, Chemiluminiscence Immunoassay. ^#^We only have data from one study made in India; a study from Greece was not included because they reported the anti-Mi-2α and anti-Mi2β prevalences without any specification of coexistence. ^&^Non-parametric tests as appropriate; *Asymptotic; **Exact Fisher test. Bold values are for highlighting the P values lower than 0.05 (significative).

**Figure 3:**
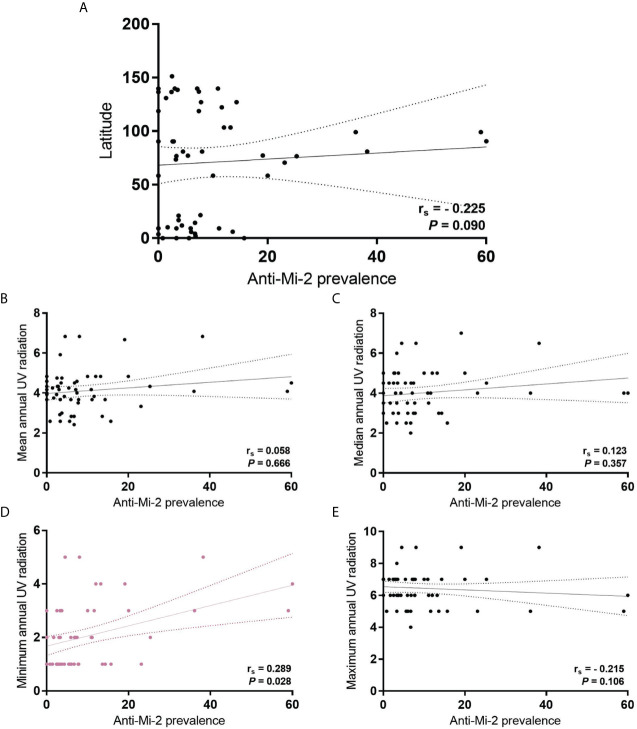
Correlation of anti-Mi-2 prevalence in all IIM subgroups with **(A)** latitude; **(B)** mean annual UV radiation; **(C)** median annual UV radiation; **(D)** minimum annual UV radiation, and; **(E)** maximum annual UV radiation.

We also looked for a correlation between MSA, MAA and UV radiation in all IIM subgroups. Regarding anti-Mi-2, we found a correlation with annual minimum UV radiation (r_s_ = 0.289, *P* = 0.028) (**Figure 3**).

### Is It a Contradiction to State That Anti-Mi-2 Does Not Associate With UV Levels but Correlates With Annual Minimum UV?

**Figure 4:**
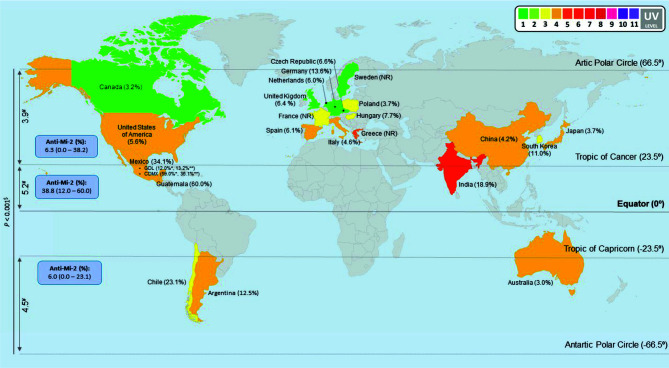
Anti-Mi-2 global prevalence in all IIM subgroups. We classified all our data per countries and according to geographic locations to obtain anti-Mi-2 prevalence. The map shows that the anti-Mi-2 prevalence increases in the geographic zone closer to the Equator and decreases in the locations farther to the Equator; likewise, UV radiation increases according to Equator proximity and it is different between geographic locations (*P* < 0.001). ^#^UV annual average radiation, we only considered the colored countries in the map. ^$^Non-parametric test, asymptotic significance. * (9). ** (6). NR: No Reported prevalence of anti-Mi-2 in these countries. Map image was made at: (https://mapchart.net/world.html).

One caveat of the studies analyzed for this review, is the lack of UV radiation real exposure data of the subjects enrolled. So, we think that in the field of environmental factors related to IIM study, it is important to consider the time and intensity to the UV exposure where the patients live. Then it is important to include this information on worldwide database. Because of the surprising behavior of our data, we decided to include the geographical latitude in this study, we re-classified the prevalence according to geographic location. We found differences between mean UV radiation of the considered countries according to the geographic location (*P* <0.001) (**Figure 4**). It is important to highlight that we also found a correlation between UV radiation and latitude, including mean (r_s_ = -0.756, *P* <0.001), median (r_s_ = -0.683, *P* <0.001), minimum (r_s_ = -0.645, *P* <0.001) and maximum (r_s_ = -0.645, *P* <0.001) (**Supplementary Figure 1**).

**Figure 5:**
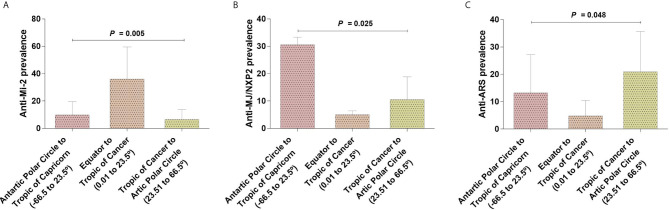
Differences of autoantibodies prevalence between geographical locations. **(A)** Anti-Mi-2 showed an increase in the geographical region closer to the Equator and a decrease in those farther to the Equator; **(B)** anti-MJ/NXP2 and **(C)** anti-ARS prevalence had an opposite behavior increasing in the geographical locations farther to the Equator.

We decided to look forward for differences in the MSA and MAA prevalence according to geographic zones instead of UV radiation level. We found differences for anti-Mi-2 (*P* = 0.005), anti-MJ/NXP2 prevalence (*P* = 0.025) and anti-ARS (*P* = 0.048) (**Table 7**, **Figure 5**). Anti-Mi-2 shows a higher prevalence in the closest region to equator (**Figure 4**).

**Table 7:** MSA and MAA prevalence according to geographic location.

MSA/MAA $\bar{x}$ (min – max)	GEOPRAPHIC LOCATION			*P^&^*
	Antarctic Polar Circle to Tropic of Capricorn (-66.5 to -23.5°)	Equator to Tropic of Cancer (0.01 to 23.5°)	Tropic of Cancer to Artic Polar Circle (23.51 to 66.5°)	
**Anti-ARS**	13.2 (3.4 – 23.1) DID-IIF-IP	4.8 (0.0 – 11.1) DID-IIF-IP	20.9 (0.0 – 54.1) ELISA, IP, ELISA-IP, IB/ELISA, DID-IIF-IP, IPB, IP-DID, LB	0.048**
- Anti Jo-1	5.8 (1.7 – 8.0) LB, MR	4.1 (0.0 – 8.3) IP-ELISA, MR	14.7 (0.0 – 48.5) DB-IIF-ELISA, DID-IP, ELISA, LB, ELISA-LB, IP, FEI, ELISA-LB-IP, ELISA-IP, DID-ELISA, ID, IP-IPB-ELISA, IPB, LB-IP, LB-IPB, MR, NR	0.053*
- Anti-PL7	1.5 (0.0 – 2.6) ELISA, LB, MR	---	4.6 (0.0 – 16.7) LB, ELISA-LB-IP, ELISA-IP, ID, IP, NR, IPB, LB-IP,	0.474**
- Anti-PL12	9.1 (1.7 – 24.0) LB, MR	---	MR 2.4 (0.0 – 9.1) ELISA-LB-IP, ELISA-IP, ID, IP, NR, IP-DID, IPB, LB,	0.266**
- Anti-EJ	---	---	LB-IP, MR 3.5 (0.0 – 30.3)	---
- Anti-OJ	---	---	IP, ELISA-LB-IP, ELISA-IP, ID, IP-DID, LB, MR 1.5 (0.0 – 6.7)	---
- Anti-KS	---	---	IP, ELISA-LB-IP, IPB, LB 2.6 (0.0 – 10.0) ELISA-IP, IP	---
**Anti-Mi-2**	9.8 (0.0 – 23.1) DID-IIF.IP, IP, LB, MR	36.1 (12.0 – 60.0) DID-IIF-IP, IP-ELISA	6.5 (0.0 – 38.2) NR, IPB, IP, DID-IIF-IP, ID, WB, ELISA, LB, IP-DID, LB-IP, LB-IPB, LB-WB, MR	0.005*
**Anti-MDA5/CADM140**	---	15.0 (0.0 – 30.0) LB	16.0 (0.0 – 67.0) ELISA, ELISA-ID, ELISA-IP-IPB, ELISA-IB, ID, IP, IPB, IP-ELISA, IP-ID-ELISA, LB, LB-ELISA, NR	0.974**
**Anti-MJ/NXP2**	30.5 (28.6 – 32.5) IP-IPB	5.0 (4.0 – 6.0) IP	10.5 (0.0 – 23.8) IPB, IP, WB, IP-DID-IPB, IP-ELISA-IPB, LB	0.025**
**Anti-TIF1α/γ (p155/140)**	13.7 (0.0 – 27.5) IP	22.0 (9.0 – 35.0) IP	15.2 (0.0 – 52.8) ELISA, IPB, ID, IP, WB, NR, LB, IP-DID	0.715**
**Anti-HMGCR**	---	---	10.4 (0.0 – 45.2) ELISA, NR, ELISA-IP, IP, LBI	---
**Anti-SAE**	---	2.0 (0.0 – 4.0) IP	2.0 (0.0 – 7.5) IPB, IP, IP-DID, ID, LB	0.830**
**Anti-SRP**	3.6 (0.0 – 12.0) DID-IIF-IP, LB	1.8 (0.0 – 6.0) DID-IIF-IP, IP	5.6 (0.0 – 42.6) LB, IP, DID-IIF-IP, ID, IPB, IP-DID, LBI, MR	0.304*
**Anti- cN1A**	34.8 ELISA	---	72.0 ELISA-WB	---
**Anti-RNP**	---	---	4.6 (0.6 – 12.4) ELISA/CI, LB, NR	---
- **Anti-U1RNP**	3.7 (0.0 – 7.5) IP	1.0 (0.0 – 2.0) IP	5.1 (0.0 – 14.9) LB, IP, ELISA-IP, DID, IPB, IP-DID	0.179**
- **Anti-U3RNP**	---	---	0.6 (0.0 – 1.9) ELISA, IP	---
- **Anti-U5RNP**	---	---	2.3 ELISA-IP	---
**Anti-Ro**	---	---	10.0 (0.0 – 44.8) DID-IP, ELISA, ELISA/CI, ID, IP, LB, IPB, IP-DID, LB-WB, NR	---
- **Anti-Ro52**	15.0 (8.7 – 29.6) ELISA, LB, MR	25.0 (21.0 – 29.0) IP-ELISA	22.3 (0.0 – 50.8) ELISA, LB, IP, LB-IPB, LB-WB	0.747**
- **Anti-Ro60**	---	17.5 (11.0 – 24.0) IP	11.7 (6.0 – 22.7) IP, LB	0.400**
**Anti-La**	---	---	1.7 (0.0 – 5.4) DID-IP, ELISA, ID, LB, IP-DID, IPB, NR	---
**Anti-PMScl**	3.7 (0.0 – 8.7) IP, MR	7.0 (2.0 – 12.0) IP	7.4 (0.0 – 31.2) IP, ID, IP-DID, LB-IPB, LB-WB	0.557**
- **Anti-PMScl75**	21.7 LB	---	6.4 (0.0 – 16.0) ELISA-IP, LB	---
- **Anti-PMScl100**	1.8 ELISA	---	3.7 (1.8 – 4.5) ELISA-IP, LB	---
**Anti-Ku**	1.7 (0.0 – 4.3) IP, LB, MR	---	3.0 (0.0 – 13.6) LB, NR, IP, ID, IP-DID, LB-IPB	0.592**
**Anti-Su**	---	---	4.6 (3.4 – 5.8) IP	---

$\bar{x}$, mean; min, minimum; max, maximum; MSA, Myositis Specific Autoantibodies; IIM, Idiopathic Inflammatory Myopathies; DM, Dermatomyositis; PM, Polymyositis; IBM, Inclusion Body Myositis; IMNM, Immune-Mediated Necrotizing Myopathy; ARS, Aminoacyl tRNA Synthetase; MDA5, Melanoma Differentiation-Associated Gene 5; NXP, Nuclear Matrix Protein; TIF1γ/α, Transcription Intermediary Factor 1γ/α; HMGCR, Hydroxymethylglutaryl coenzyme A reductase; SAE, Small ubiquitin-like modifier Activating Enzyme; SRP, Signal Recognition Particle; cN1A, cytosolic 5’ nucleotidase 1A; RNP, ribonucleoprotein; DID, Ouchterlony Double Immunodifussion; IIF ,Indirect Immunofluorescence; IP ,Immunoprecipitation; DB, Dotblot; ELISA, Enzyme-Linked Immunosorbant Assay; FEI, Fluorescence Enzyme Immunoassay; MR, Medical Records; IPB, Immunoprecipitation-blotting; NR ,No Reported; WB ,Western Blot; LBI, Laser Bead Immunoassay; IB, Immunoblot; CI, Chemiluminiscence Immunoassay. ^&^Non-parametric tests as appropriate; *Asymptotic; **Exact Fisher test. Bold values are for highlighting the P values lower than 0.05 (significative).

Although we found differences, the data behavior was not the same; for example, in the case of anti-Mi-2, we observed that the prevalence increased in the region closer to the Equator and decreased in the other two geographic locations (**Figures 4** and **5**). In the case of anti-MJ/NXP2 we observed a major prevalence in the region from Antarctic Polar Circle to Tropic of Capricorn and this decreased while approaching to Equator (**Figure 5**) while anti-ARS prevalence increases in the zone from Tropic of Cancer to Artic Polar Circle (**Figure 5**).

### Anti-Mi-2, Anti-PL12, Anti-Ro52 and Anti-PMScl-75 Autoantibodies Have a Negative Correlation With Geographical Latitude

**Figure 6:**
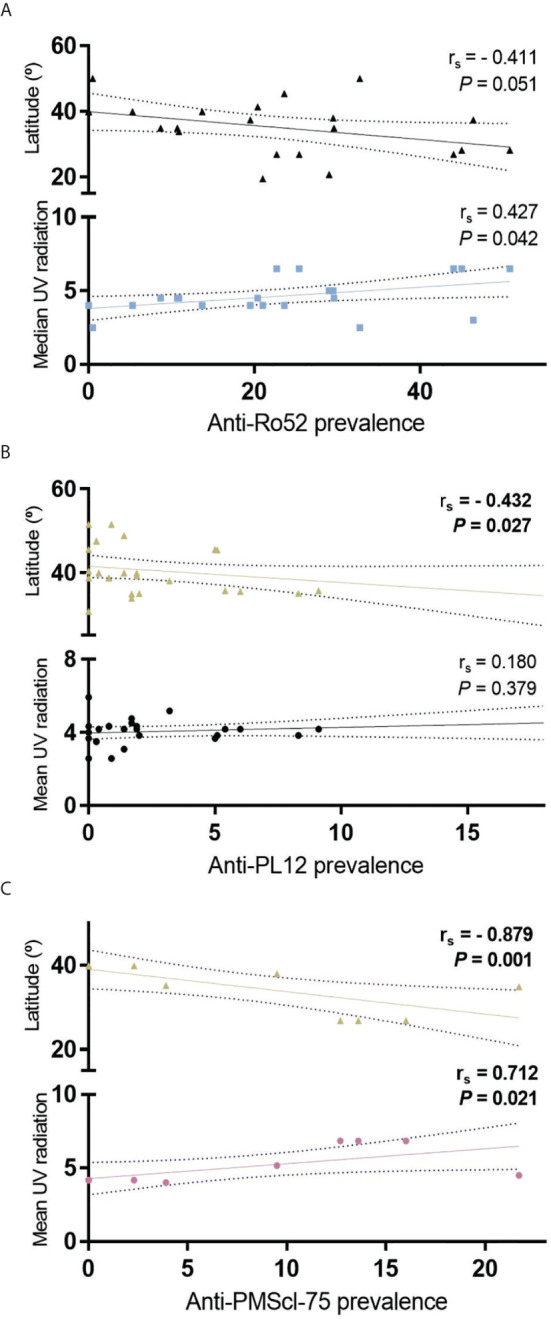
Correlation of geographical latitude and mean UV radiation with **(A)** anti-Ro52; **(B)** anti-PL12 and; **(C)** anti-PMScl-75 in all IIM subgroups.

We investigated the correlation between latitude and autoantibodies prevalence and we observed that anti-Mi-2 (**Figure 4A**), and anti-Ro-52 (**Figure 6A**) prevalence follows a trend to diminish while latitude increases. The prevalence of anti-PL12 and anti-PMScl-75 have a negative correlation with geographical latitude (**Figures 6B, C** **)**. However, only anti-PMScl-75 autoantibody also showed a correlation with mean UV radiation (**Figure 6C**).

## Discussion

UV radiation intensity changes every day, for this reason its measure becomes complicated. In this systemic review, we tried to obtain an UV annual approximate of every city where autoantibodies prevalence was reported; however, the time period of patient recruitment was very wide (even up to 10 years). Since these difficulties, we could not observe a direct correlation between UV radiation and MSA. In order to get better results, we encourage to consider the UV radiation levels in future MSA and MAA prevalence reports.

Because of this situation, our alternative option was to consider geographic latitude; it gave us greater precision in our analysis. Additionally, we successfully achieved to show a correlation between latitude and UV radiation.

Our main results revealed that anti-PL7, anti-Ro52, anti-La and anti-Ku showed differences between UV radiation levels. Once we analyzed the prevalence according to latitude, we could observe a difference of anti-Mi-2 prevalence according to the geographical zone as well as a trend to a correlation between anti-Mi-2 prevalence and proximity to the Equator.

Interestingly, we noticed differences in the prevalence of anti-ARS and anti-MJ/NXP2 between the geographical locations, but they did not correlate with geographical latitude. In the case of anti-Mi-2, it was different between geographic zones and showed a trend to diminish in higher latitude.

Otherwise, we found that the prevalence of anti-PL12, and anti-PMScl-75 are correlated to geographical latitude. It is important to note that only anti-PMScl-75 correlated with both geographical latitude and UV radiation, might due to the lack of UV radiation accurate data.

The behavior of these data suggests that the prevalence of anti-PL12, anti-Ro52 and anti-PMScl-75 autoantibodies also increases according to the equator proximity. Anti-Ro52, had already been previously reported to follow this behavior (11); however, to our knowledge, this is the first study reporting the differential geographic distribution of anti-PL12 and anti-PMScl-75.

Although the mechanisms that regulate this behavior and geographical distribution remain unclear, they could be associated to UV radiation as a key factor in the pathogenesis of the disease. In addition, it has been documented that the source of autoantigens in other rheumatic autoimmune diseases such as Systemic Lupus Erythematosus (SLE), comes from debris released after apoptosis including Ro52, Ro60, La, U1, and even Mi-2 (109). It is important to highlight that we could observe an increment in autoantibodies against Mi-2 and Ro-52 according to their proximity to the Equator and higher UV radiation levels. This probably could be related with UV radiation.

Although we initially considered 242 articles, we only obtained relevant information of 92; however, there is still a poorly information and we had limitations such a lack of reports about prevalence of all the autoantibodies of interest in different countries and UV levels in order to get a better overview of the behavior of MSAs and MAAs according to geographical location and the role of UV radiation in the development of autoimmunity.

In this review we could obtained MSA and MAA prevalence data from 22 countries and we offer the evidence that there are differences in their prevalence between countries, as well as the fact that anti-Jo-1 is not the most prevalent autoantibody around the world, just in European countries. In Mexico, anti-Mi-2 is the most prevalent autoantibody reported maybe to UV radiation; however, other environment factors need to be considered. In summary, in the case of anti-Mi-2 we observed that the prevalence increased in the region closer to the Equator, meanwhile anti-MJ/NXP2 and anti-ARS demonstrated a major prevalence far from Equator zone.

## Limitations 

UV radiation intensity changes every day, for this reason its measure becomes complicated. In this systemic review, we tried to obtain an UV annual approximate of every city where autoantibodies prevalence was reported; however, the time period of patient recruitment was very wide (even up to 10 years). Since these difficulties, we could not observe a direct correlation between UV radiation and anti-MSA.

## Data Availability Statement

The original contributions presented in the study are included in the article/**Supplementary Material**. Further inquiries can be directed to the corresponding author.

## Author Contributions

AA-V. Contributed to the conception and design of the study, carried out the statistics and participated in analysis and interpretation of data. Drafted the paper and approved the final version of the manuscript. EC-A. Contributed to the conception and design of the study, carried out the statistics and participated in analysis and interpretation of data. Drafted the paper and approved the final version of the manuscript. OP-M. Contributed to the execution and participated in analysis and interpretation of data. Critically revised the draft of article and approved the final version of manuscript. LA-O. Contributed to the execution and participated in analysis and interpretation of data. Critically revised the draft of article and approved the final version of manuscript. AR-H. Contributed to the execution, carried out the statistics and participated in data interpretation. Critically revised the draft of article and approved the final version of manuscript. E-DR-A. Contributed to the execution, carried out the statistics and participated in data interpretation. Critically revised the draft of article and approved the final version of manuscript. MV-M. Contributed to the conception and the design of the study, execution, verified the analysis and interpretation of data. Critically revised the draft of article and approved of the final version of manuscript. All authors contributed to the article and approved the submitted version.

## Funding

This work was funded by Fondo de Desarrollo Cientifico (FODECIJAL) 2019 from Consejo Estatal de Ciencia y Tecnología de Jalisco (COECYTJAL), with approval number 1702512-8152.

## Conflict of Interest

The authors declare that the research was conducted in the absence of any commercial or financial relationships that could be construed as a potential conflict of interest.

## Supplementary Material

Supplementary Figure 1 Negative correlation of geographic latitude with **(A)** mean annual UV radiation; **(B)** median annual UV radiation; **(C)** minimum annual UV radiation, and; **(D)** maximum annual UV radiation.
