# Supplementary figures and images for: Geographical Latitude Remains as an Important Factor for the Prevalence of Some Myositis Autoantibodies: A Systematic Review
Source: Front Immunol. 2021 Apr 22;12:672008. doi: 10.3389/fimmu.2021.672008 (PMC8100663; doi:10.3389/fimmu.2021.672008)

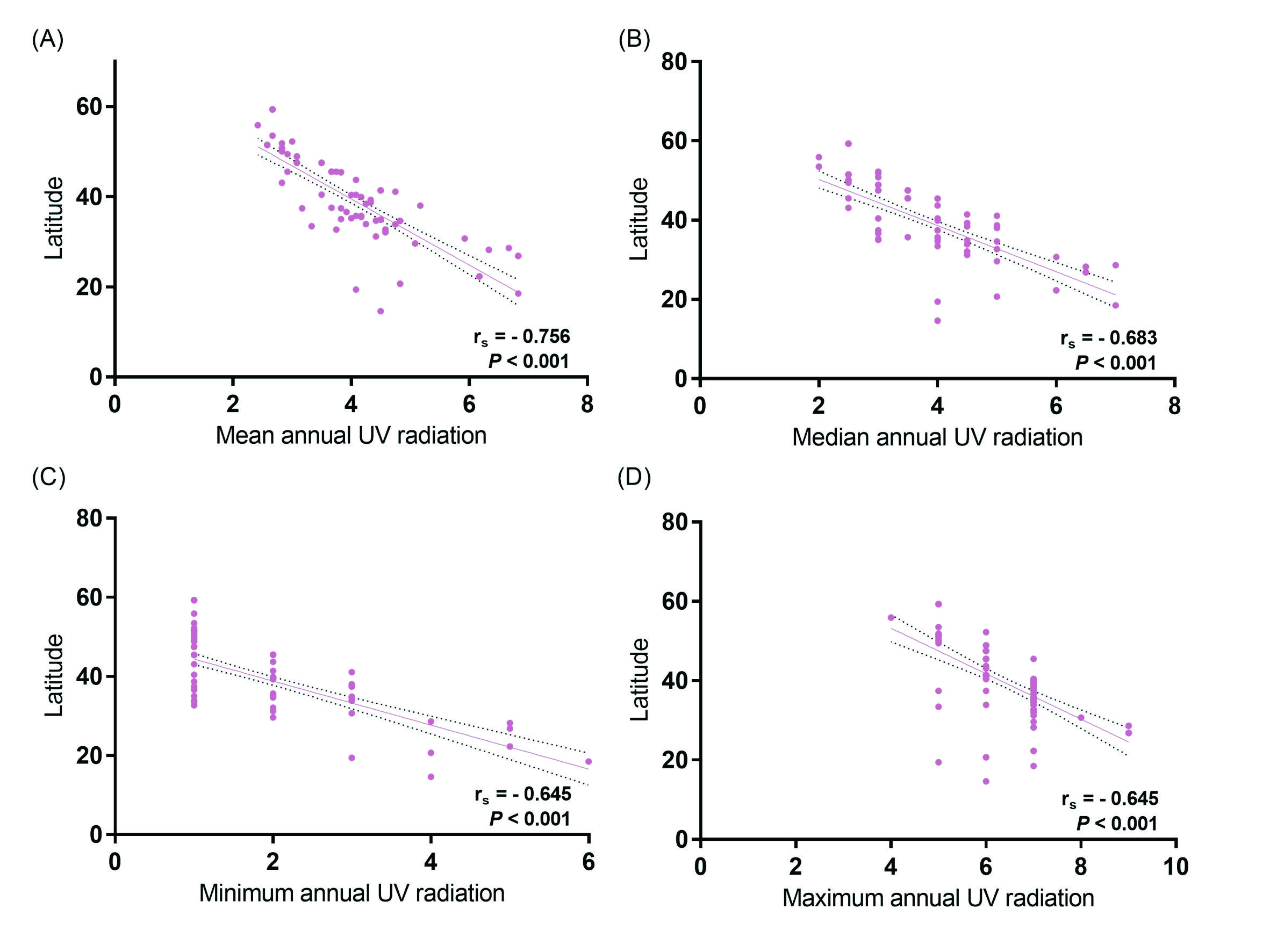

Supplement: Supplementary Figure 1 — Negative correlation of geographic latitude with (A) mean annual UV radiation; (B) median annual UV radiation; (C) minimum annual UV radiation, and; (D) maximum annual UV radiation. [file Image_1.jpeg]
