# Supplementary material for: Geographical Latitude Remains as an Important Factor for the Prevalence of Some Myositis Autoantibodies: A Systematic Review
Source: Front Immunol. 2021 Apr 22;12:672008. doi: 10.3389/fimmu.2021.672008 (PMC8100663; doi:10.3389/fimmu.2021.672008)
Supplement: Supplementary file 2 [file Table_1.docx]

**Supplementary Table 1. Reports Summary included in the Systematic Review.**

| N° | Author | Objectives | Subjects (n) | | Location | Method | | Results  (autoantibodies prevalences and frequencies) | |
| --- | --- | --- | --- | --- | --- | --- | --- | --- | --- |
| 1 | Temmoku 2019 | To analyze the phenotype and clinical outcomes of patients with PM/DM regarding the association of anti-MDA5 or anti-ARS Abs, and also to assess the value of anti-SSA/Ro52 Ab as a prognostic marker. | 84 patients with PM/DM | | Koriyama, Fukushima Japan | ELISA by MESACUP | | Anti-MDA5: 18/84 (21.4%)  Anti-ARS: 31/84 (36.9%)  Anti-Ro52: 39/84 (46.4%)  Anti-Jo-1: 17/84(20.2%) | |
| 2 | Szabó 2018 | It does not report autoantibodies prevalences | | | | | | | |
| 3 | Huang 2018 | To detect anti-HMGCR antibody in Chinese patients with myositis and to clarify the features of anti-HMGCR-positive patients. | 181 adult Chinese DM/PM patients. | | Changsha City, Hunan, China. | Immunoprecipitation (anti-HMGCR) and Line-blot by EUROIMMUNE (Anti-Jo-1, anti-Ro52, anti-SSA/Ro, anti-SSB/La) | | Anti-HMGCR: 12/181(6.6%)  Anti-Jo-1: 33/181 (18.2%)  Anti-Ro-52: 92/181 (50.8%)  Anti-SSA/Ro: 29/181 (16.2%)  Anti-SSB/La: 4/181 (2.2%) | |
| 4 | Best 2018 | It does not report autoantibodies prevalences | | | | | | | |
| 5 | Hoesly 2018 | It does not report autoantibodies prevalences | | | | | | | |
| 6 | Ibañez 2018 | To determine potential prognostic factors in different types of IIM associated with ILD through analysis of mortality, the need for long-term oxygen therapy (LTOT), and deteriorations in respiratory function tests. | 478 Patients with IIM, (DM, PM, Overlap myositis, Anti-synthetase syndrome) | | Madrid, Madrid, Spain | Immunoblot or ELISA (anti-Jo-1, anti-PL7, anti-PL12), ELISA or chemiluminiscence immunoassay (anti-RNP, anti-SSA/Ro). | | Anti-ARS: (19.3%)  Anti-RNP: (12.4%)  Anti-SSA/Ro: (12.1%) | |
| 7 | Huber 2018 | It does not report autoantibodies prevalences | | | | | | | |
| 8 | Jiao 2018 | To clarify the phenotypes and therapeutic responses of statin-naïve anti-HMGCR antibody-mediated necrotizing myopathy | 98 PM, DM, nonspecific myositis and sIBM, or fulfilled the revised criteria of IMNM | | Shangai, Shanghai, China | ELISA by Costar | | Anti-HMGCR: 21/98 (21.4%) | |
| 9 | Zampeli 2018 | To detect the prevalence and possible clinical associations of MSAs and MAAs in a cohort  of Greek IIM patients who were classified based on the latest  IIM criteria | 95 IIM patients | | Athens, Attica, Greece, | Line-blot by EUROIMMUNE | | Anti-Ro-52: 30/95 (31.6%)  Anti-Jo-1: 21/95 (22.1%)  Anti-SRP: 12/95 (12.6%)  Anti-PMScl-75: 9/95 (9.5%)  Anti-TIF1γ: 7/95 (7.4%)  Anti-Mi-2α: 6/95 (6.3%)  Anti-Mi-2β: 6/95 (6.3%)  Anti-SAE1: 6/95 (6.3%)  Anti-Ku: 6/95 (6.3%)  Anti-NXP2: 4/95 (4.2%)  Anti-PMScl-100: 4/95 (4.2%)  Anti-PL-12: 3/95 (3.2%)  Anti-PL-7: 1/95 (1.0%)  Anti-MDA5: 1/95 (1.0%)  Anti-EJ: 1/95 (1.0%)  Anti-OJ: 1/95 (1.0%)  Anti-ARS: 2/95 (2.1%) | |
| 10 | Touat 2018 | It does not report autoantibodies prevalences | | | | | | | |
| 11 | Takada 2018 | It does not report autoantibodies prevalences | | | | | | | |
| 12 | Wang 2018 | To identify a subset of patients with myositis carrying anti-NXP2 antibodies | 120 Chinese adult patients, of definite or probable PM/DM | | Changsan city, Hunan, China | Immunoprecipitation (anti-NXP2), line-blot by EUROIMMUNE ( anti-Jo-1, anti-SSA/Ro, anti-Ro-52, anti-SSB/La, anti-PMScl-70) | | Anti-NXP-2: 10/120 (8.3%)  Anti-Jo-1: 23/120 (19.2%)  Anti-SSA/Ro: 21/120 (17.5%)  Anti-Ro-52: 54 /120 (45%)  Anti-SSB/La: 2/120 (1.7%) | |
| 13 | Endo 2018 | It does not report autoantibodies prevalences | | | | | | | |
| 14 | Hinze 2018 | It does not report autoantibodies prevalences | | | | | | | |
| 15 | Rietveld 2018 | It does not address Idiopathic Inflammatory Myopathies | | | | | | | |
| 16 | Ogawa 2018 | It does not report autoantibodies prevalences | | | | | | | |
| 17 | Wang 2018 | It does not report autoantibodies prevalences | | | | | | | |
| 18 | Pachman 2018 | Review | | | | | | | |
| 19 | Ueki 2018 | To clarify the association of MSAs with clinical and laboratory features in Japanese patients with juvenile idiopathic inflammatory  myopathies | 25 Japanese patients with Juvenile idiopathic Inflammatory myopathies | | Sapporo, Hokkaida, Japan. | ELISA (anti-MDA5), RNA-immunoprecipitation (anti-ARS, anti-SRP, anto-U1RNP, anti-SSA/Ro, anti-SSB/La), immunoprecipitation-immunoblotting (anti-Mi-2, anti-TIF-1γ, anti-MJ/NXP2, anti-SAE, anti-Ku), fluorescence enzyme immunoassay (anti-Jo-1), not defined (anti-HMGCR) | | Anti-MDA5: 7/25 (28%)  Anti-TIF-1γ: 5/25 (20%)  Anti-MJ/NXP-2: 4/25 (16%)  Anti-Jo-1: 2/25 (8%)  Anti-HMGCR: 1/25 (4%)  Anti-SRP: 1/25 (4%) | |
| 20 | So 2018 | To compare the prevalence of the anti-MDA5 antibody | 20 patients with DM | | Hong Kong, Hong Kong, China | Line-blot by EUROIMMUNE | | DM:  Anti-MDA5: 6/20 (30%)  PM:  Anti-MDA5: 0/20 (0%) | |
| 21 | Deakin 2018 | It does not report autoantibodies prevalences | | | | | | | |
| 22 | Chen 2018 | To compare the heterogeneity of interstitial lung disease (ILD) in patients with PM/DM according to serological type | 182 patients with PM/DM-ILD | | Beijing, Beijing, China | Line-blot by EUROIMMUNE | | Anti-MDA5: 43/182 (23.6%)  Anti-ARS: 56/182 (30.8%). | |
| 23 | Lundberg 2018 | It does not report autoantibodies prevalences | | | | | | | |
| 24 | Yura 2017 | To clarify the clinical characteristics of patients with anti-ARS antibody positive by comparing those without anti-ARS antibodies or PM/DM-ILD with anti-ARS antibodies. | 37 patients PM/DM-ILD | | Nagasaki, Nagasaki, Japan | Immunoprecipitation | | Anti-ARS: 20/37 (54.1%)  Anti-Jo-1: 9/37 (24.3%)  Anti-KS: 4/37 (10%)  Anti-PL-7: 4/37 (10.8%) | |
| 25 | Yang 2017 | To systematically define the cancer-associated MSAs in IIMs. | 72 patients with malignancies related to IIMs | | Beijing, Beijing, China | Line-blot by EUROIMMUNE, ELISA by INOVA Diagnostics (anti-HMGCR) | | Anti-TIF1-γ: 38/72 (52.8%)  Anti-NXP2: 3/72 (4.2%) Anti-SAE1: 4/72 (5.5%) Anti-ARS: 10/72 (14%) Anti-Jo-1: 5/72 (7%)  Anti-PL-12: 1/72 (1.4%) Anti-PL-7: 1/72 (1.4%)  Anti-EJ: 1/72 (1.4%) Anti-MDA5: 1/72 (1.4%) | |
| 26 | Malaviya 2017 | Letter to the autor | | | | | | | |
| 27 | Lin 2017 | To understand the contribution of HLA II genetic variants to DM, | 224 DM patients  . | | Beijing, Beijing China | Line-blot by EUROIMMUNE | | Anti-ARS: 36/224 (16.1%)  Anti-Jo-1: 19/224 (8.5%)  Anti-EJ: 5/224 (2.2%)  Anti-PL-7: 11/224(4.9%)  Anti-PL-12: 1/224 (0.4%)  Anti-MDA5: 36/224 (16.1%) | |
| 28 | Mainetti 2017 | Review | | | | | | | |
| 29 | Danielsson 2017 | It does not report autoantibodies prevalences | | | | | | | |
| 30 | Kim 2017 | It does not report autoantibodies prevalences | | | | | | | |
| 31 | Suzuki 2017 | It does not report autoantibodies prevalences | | | | | | | |
| 32 | González 2017 | It does not report autoantibodies prevalences | | | | | | | |
| 33 | Ogawa 2017l | To establish a simple method for screening anti-Su/Ago2 antibodies and to analyze clinical features associated with DM/PM patients with these antibodies. | 224 Patients with DM/PM | | Kitakyushu, Fukuoka, Japan | ELISA by MBL (anti-Ro60, anti-U1RNP, anti-Mi-2, anti-TIF1γ, anti-MDA-5, anti-NXP-2, anti-HMG-CoA, anti-Ku70/80, anti SRP54, anti-PM/Scl-75/100 and anti-SAE1/2) and in-house (anti-EJ, anti-Jo-1, anti-KS, anti-PL7, anti-PL12), and immunoprecipitation (anti-Su/Ago2) | | Anti-Su/Ago2: 13/224 (5.8%)  Anti-MDA5: 53/224 (23.7%)  TIF-1γ: 37/224 (16.5%)  Anti-ARS: 35/224 (15.6%) | |
| 34 | Tansley 2017 | Multicentric study, it does not report autoantibodies prevalences per city | | | | | | | |
| 35 | Spencer 2017 | It does not report autoantibodies prevalences | | | | | | | |
| 36 | Tokunaga 2017 | Case report | | | | | | | |
| 37 | Fujiki 2017 | It does not report autoantibodies prevalences | | | | | | | |
| 38 | Shi 2017 | To compare the clinical characteristics and to identify the longterm outcomes of Chinese patients with different antisynthetase antibodies. | 639 IIM patients | | Beijing, Beijing, China | Line-blot by EUROIMMUNE | | Anti-ARS: 124/639 (19.4%)  Anti-Jo-1: 62/639 (9.7%) Anti-PL-7: 31/639 (4.9%) Anti-PL-12: 12/639 (1.9%) Anti-EJ: 19/639 (3%)  Anti-SRP: 2/639 (0.3%) Anti-MDA5: 2/639(0.3%)  Anti-RNP: 5/639 (0.8%)  Anti-Ro60: 38/639 (6%)  Anti-Ro52: 34/639 (5.3%) | |
| 39 | Tanizawa 2017 | To elucidate the long-term outcome of ARS-ILD with and without PM/DM. | 51 DM/PM+ ILD patients | | Kyoto, Kyoto, Japan | RNA-immunoprecipitation | | Anti-Jo-1: 9/51 (17.6%) Anti-PL-7: 4/51 (7.8%)  Anti-EJ: 3/51 (5.9%)  Anti-PL12: 1/51 (2%) | |
| 40 | Rider 2017 | It does not report autoantibodies prevalences | | | | | | | |
| 41 | Pinal-Fernandez 2017 | To study the prevalence, rate of appearance and severity of clinical features in patients with different anti-synthetase syndrome autoantibodies. | 1198 IIM patients | | Baltimore, Maryland, USA | ELISA, Line-blot by EUROIMMUNE and Immunoprecipitation  All the autoantibodies were measured by the three methods | | Anti-Jo-1: 124/1198 (10.3%)  Anti-PL12: 23/1198 (1.9%)  Anti-PL7: 16/1198 (1.3%) Anti-EJ: 3/1198 (0.3%)  Anti-OJ: 3/1198 (0.3%) | |
| 42 | Hussain 2017 | To determine autoantibody profile in children with JDM. | 30 JDM patients | | Chandigarh, Chandigarh, India | Immunodot kit, Blue Dot PMS12-24 from D-tek Belgium. | | Anti-PL-7: 0/30 (0%)  Anti-PL-12: 0/30 (0%) Anti-EJ: 0/30 (0%)  Anti-Ku: 0/30 (0%)  Anti-Scl-70: 0/30 (0%) Anti-SSA/Ro: 0/30 (0%)  Anti-SRP: 3/30 (10%) Anti-MDA5: 2/30 (6.7%)  Anti-Jo1: 1/30 (3.3%)  Anti-TIF1-γ: 1/30 (3.3%) Anti-Mi-2: 1/30 (3.3%)  Anti-PM-Scl: 1/30 (3.3%) | |
| 43 | Kuye 2017 | Paper was not accessed | | | | | | | |
| 44 | Ge 2017 | To analyze the clinical features of anti-SAE antibodies in Chinese myositis patients in comparison with different cohorts. | 538 PM /DM patients  394 DM and 144 PM | | Beijing, Beijing,China | Immunoprecipitation | | PM/DM:  Anti-SAE: 12/538 (2.2%)  DM:  Anti-SAE: 12/394 (3.0%)  PM:  Anti-SAE: 0/144 (0%) | |
| 45 | De souza 2017 | Paper was not accessed | | | | | | | |
| 46 | Sarkar 2017 | It does not report autoantibodies prevalences | | | | | | | |
| 47 | Limaye 2017 | To characterize the role of vaccinations and infections in triggering idiopathic inflammatory myopathies | 115 IIM patients | | Adelaide, South Australia, Australia | Medical records | | Anti-Ro-52: 34/115 (29.6%) Anti-Jo-1: 9/115 (7.8%) Anti-Mi-2: 4/115 (3.5%) Anti-PL-7: 3/115 (2.6%) Anti-PL-12: 2/115 (1.7%)  Anti-PMScl: 10/115 (8.7%) Anti-Ku: 3/115 (2.6%) | |
| 48 | Kohara 2017 | Case report | | | | | | | |
| 49 | Mecoli 2017 | It does not report autoantibodies prevalences | | | | | | | |
| 50 | Rogers 2017 | To characterize the cutaneous and systemic clinical phenotype of DM patients with anti–NXP2 antibodies | 178 DM patients | | Baltimore, Maryland, USA | Immunoprecipitation and ELISA (anti–Jo-1, anti–Ro52) | | Anti-NXP-2: 20/178 (11.2%) | |
| 51 | Albayda 2017 | To characterise the clinical features associated with anti–NXP-2 autoantibodies | 235 patients with DM | | Bethesda, Maryland, USA | Immunoprecipitation | | Anti–NXP-2: 56/235 (23.8%)  Anti-TIF1-γ: 26/99 (26.3%) Anti-Mi-2: 25/99 (25.3%) Anti-Jo-1: 22/99 (22.2%) Anti-PMScl: 13/99 (13.1%) Anti-MDA5: 7/99 (7.1%) | |
| 52 | Foreman 2017 | It does not report autoantibodies prevalences | | | | | | | |
| 53 | Svensson 2017 | To characterize the Swedish IIM patients who have been treated with biologics | 95 IIM patients:  31 PM  27 DM  8 JDM  29 IBM | | Stockholm, Sweden | Medical records | | IIM: Anti-Jo-1: 17/95 (17.9%) PM: Anti-Jo-1: 8/31 (25.8%)  DM: Anti-Jo-1: 9/27 (33.3%)  JDM: Anti-Jo-1: 0/8 (0%)  IBM: Anti-Jo-1: 0/29 (0%) | |
| 54 | Nawata 2016 | It does not report autoantibodies prevalences | | | | | | | |
| 55 | Fredi 2016 | Paper was not accessed | | | | | | | |
| 56 | Sasano 2016 | It does not report autoantibodies prevalences | | | | | | | |
| 57 | Tall 2016 | It does not address Idiopathic Inflammatory Myopathies | | | | | | | |
| 58 | Tampoia 2016 | To characterize the autoantibody profile in a cohort of Italian patients with IIM using two new commercially available myositis profiling assays | 53 IIM adult Caucasian | | Bari, Puglia, Italy. | Immunodot by Sclero-Poly-Synthetase Profile 12 DOT (anti-Jo1, anti-PL-7, anti-PL-12, anti-EJ, anti-SRP-54, anti-Mi-2, anti-MDA5, anti-TIF1γ, anti-Ku, anti-PM-Scl 100, anti-Scl-70, and anti-SSA/Ro52) and by BlueDot Polymyositis IgG (anti-OJ, anti-KS, anti-HA, anti-ZO, anti-NXP2, anti-SAE-1, SAE-2, anti-EIF-3, anti-HMGCR, anti-RNAP-III) from Alphadia | | Anti-Jo-1: 13/53 (24.5)  Anti-TIF1γ: 1/53 (1.8%)  Anti-Mi-2: 2/53 (3.7%)  Anti- SAE: 4/53 (7.5%) | |
| 59 | Tan 2016 | To review the use of a line immunoassay-based myositis panel incorporating both MSA and myositis-associated autoantibodies (MAA) in a selected population of patients | 119 IIM patients | | Sydney, New South Wales, Australia | Line-blot by EUROIMMUNE | | Anti-ARS: 4/119 (3.4%) Anti-Jo-1: 2/119 (1.7%) Anti PL-12: 2/119 (1.7%) Anti-SRP: 3/119 (2.5%)  Anti-Mi2: 3/119 (2.5%)  Anti Ro-52: 13/119 (10.9%) | |
| 60 | Deakin 2016 | To investigate whether histopathologic  findings and MSAs have prognostic significance in juvenile DM. | 101 patients with JDM | | London, London, UK | Immunoprecipitation and ELISA (anti-NXP2 and anti-MDA5) | | Anti–TIF-1γ: 18/90 (20%)  Anti–NXP-2: 15/90 (16.7%)  Anti-MDA5: 11/90 (12.2%)  Anti–Mi-2: 5/90 (5.6%)  Anti-SRP: 2/90 (2.2%)  Anti-PL-7: 1/90 (1.1%) Anti-SAE: 1/90 (1.1%) Anti-PM-Scl: 6/90 (6.7%) Anti-U1RNP 2/90 (2.2%) | |
| 61 | Ceribelli 2016 | to characterize MSAs in a well-defined cohort of patients with IIM and to determine their association with cancer | 38 patients with PM/DM  18 DM  20 PM | | Milan, Italy | Immunoprecipitation | | PM/DM  Anti–TIF-1γ: 4/38 (10.5%)  Anti–NXP-2: 3/38 (7.9%)  Anti–Mi-2: 2/38 (5.2%)  Anti-SRP: 2/38 (5.2%)  Anti-Jo-1: 4/38 (10.6%) Anti-EJ: 1/38 (2.6%)  Anti-PL-12: 1/38 (2.6%) Anti-HMGCR: 0/38 (0%)  Anti-MDA5: 0/38 (0%)  DM  Anti–TIF-1γ: 4/18 (22.2%)  Anti–NXP-2: 3/18 (16.7%)  Anti–Mi-2: 2/18 (11.1%)  Anti-SRP: 1/18 (5.6%)  Anti-Jo-1: 0/18 (0%) Anti-EJ: 0/18 (0%)  Anti-PL-12: 0/18 (0%) Anti-HMGCR: 0/18 (0%)  Anti-MDA5: 0/18 (0%)  PM  Anti–TIF-1γ: 0/20 (0%)  Anti–NXP-2: 0/20 (0%)  Anti–Mi-2: 0/20 (0%)  Anti-SRP: 1/20 (5%)  Anti-Jo-1: 4/20 (2%) Anti-EJ: 1/20 (5%)  Anti-PL-12: 1/20 (5%) Anti-HMGCR: 0/20 (0%)  Anti-MDA5: 0/20 (0%) | |
| 62 | Guffroy 2016 | It does not address Idiopathic Inflammatory Myopathies | | | | | | | |
| 63 | Hoa 2016 | It does not address Idiopathic Inflammatory Myopathies | | | | | | | |
| 64 | Zamora 2016 | It does not report autoantibodies prevalences | | | | | | | |
| 65 | Belutti 2016 | It does not report autoantibodies prevalences | | | | | | | |
| 66 | Martel 2016 | It does not report autoantibodies prevalences | | | | | | | |
| 67 | Kobayashi 2016 | It does not address Idiopathic Inflammatory Myopathies | | | | | | | |
| 68 | Yoshida  2016 | Case report | | | | | | | |
| 69 | Gomez 2016 | To explore the frequency of MSAs and MAAs and their relation­ship with clinical features in adult patients with idiopathic inflammatory myopathies. | 25 IIM patients | | Buenos Aires, Argentina | Line-blot by INNO-LIA ANA update 28662 v1 (anti-Ro, anti-La, anti-ribosomal-P, anti-RNP, anti-Sm, anti-centromere and anti-topoisomerase) and by Myositis plus ORG 760, Orgentec Diagnostika GmbH (anti-Mi-2, anti-Jo-1, anti-PM-Scl-100, antiPL-7, anti-PL-12, anti-Ku, and anti-SRP) | | Anti-Mi-2: 5/25 (20%) Anti-PL-12: 6/25 (24%) Anti-PL-7: 0/25 (0%) Anti-Jo-1: 2/25 (8%)  Anti-SRP: 3/25 (12%) | |
| 70 | Hetlevik 2016 | It does not report autoantibodies prevalences | | | | | | | |
| 71 | Srivastava 2016 | To study the prevalence and clinical associations MSAs and MAAs in a large cohort of Indian patients with IIM. | 102 PM/DM/JDM patients  80 PM/DM patients  55 DM patients  25 PM patients  22 JDM patients | | Lucknow, Uttar Pradesh, India | Line-blot by EUROIMMUNE | | PM/DM/JDM patients  Anti-Mi-2: 25/102 (24.5%)  Anti-SRP: 5/102 (4.9%)  Anti-Jo-1: 14/102 (13.7%)  Anti-ARS: 13/102 (12.7%) Anti-Ro52: 30/102 (29.4%) Anti-Ku: 11/102 (10.8%) Anti-PM-Scl100: 3/102 (2.9%)  Anti-PM-Scl75: 10/102 (9.8 %)  80 PM/DM patients  Anti-Mi-2: 23/80 (28.7%)  Anti-SRP: 4/80 (5%)  Anti-Jo-1: 9/80 (11.2%)  Anti-ARS: 10/80 (12.5%) Anti-Ro52: 25/80 (31.2%) Anti-Ku: 8/80 (10%) Anti-PM-Scl100: 2/80 (2.5%)  Anti-PM-Scl75: 7/80 (8.7%)  55 DM patients  Anti-Mi-2: 21/55 (38.2%)  Anti-SRP: 3/55 (5.5%)  Anti-Jo-1: 3/55 (5.5%)  Anti-ARS: 7/55 (12.7%) Anti-Ro52: 14/55 (25.4%) Anti-Ku: 7/55 (12.7%) Anti-PM-Scl100: 1/55 (1.8%)  Anti-PM-Scl75: 7/55 (12.7%)  25 PM patients  Anti-Mi-2: 2/25 (8%)  Anti-SRP: 1/25 (4%)  Anti-Jo-1: 6/25 (24%)  Anti-ARS: 3/25 (12%) Anti-Ro52: 11/25 (44%) Anti-Ku: 1/25 (4%) Anti-PM-Scl100: 1/25 (4%)  Anti-PM-Scl75: 4/25 (16%)  22 JDM patients  Anti-Mi-2: 1/22 (4.5%)  Anti-SRP: 1/22(4.5%)  Anti-Jo-1: 5/22 (22.7%)  Anti-ARS: 3/22 (13.6%) Anti-Ro52: 5/22 (22.7%) Anti-Ku: 3/22 (13.6%) Anti-PM-Scl100: 1/22 (4.5%)  Anti-PM-Scl75: 3/22 (13.6%) | |
| 72 | Kennedy 2016 | It does not report autoantibodies prevalences | | | | | | | |
| 73 | Garber 2016 | It does not address Idiopathic Inflammatory Myopathies | | | | | | | |
| 74 | Pinal-Fernandez 2016 | To identify factors associated with disease severity and clinical improvement and to compare the severity of weakness in those with anti-SRP versus anti-HMGCR autoantibodies. | 732 IIM patients | | Baltimore, Maryland, USA | Immunoprecipitation | | Anti-SRP: 39/732 (5.3%) | |
| 75 | Allenbach 2016 | To screen the incidence of cancer in necrotizing autoimmune myopathies. | 115 IMNM patients | | Paris, Ile-de-France, France | Laser bead immunoassay (anti-HMGCR, anti-SRP) and Line-blot by D-tek (anti-Jo-1, anti-PL7, anti-PL12, anti-Mi-2, anti-MDA5) and EUROIMMUNE (anti-NXP2, anti-TIF-1γ) | | Anti-HMGCR: 52/115 (45.2%)  Anti-SRP: 49/115 (42.6%) | |
| 76 | Kawasumi 2016 | To evaluate the present status of physical dysfunction and to clarify the clinical manifestations and (MSAs) associated with physical dysfunction after treatment in PM/DM. | 77 PM/DM patients | | Tokyo, Tokyo, Japan | Immunoprecipitation (anti-ARS, anti-SRP, anti-Ku, anti-SSA/Ro), ELISA (anti-MDA5), Western-blot (anti-TIF1-𝛾, anti-Mi-2, anti-NXP-2) and Ouchterlony double immunodiffusion assay (anti-U1RNP) | | Anti-ARS: 22/67 (32.8%)  Anti-MDA5: 7/67 (9.6%)  Anti-Mi-2: 2/67 (3%)  Anti-NXP-2: 2/67(3%)  Anti-SRP: 9/67(13.4%)  Anti-TIF1-𝛾: 4/67 (6%)  Anti-Ku: 3/67 (4.5%)  Anti-U1RNP: 10/67 (14.9%)  Anti SSA: 30/67(44.8%) | |
| 77 | Bodoki 2016 | To highlight rare associations of myositis specific autoantibodies | 337 PM/DM patients | | Debrecen, Debrecen, Hungary | Line-blot by Orgentec Diagnostika (anti-Jo-1, anti-PL-7, anti-PL-12, anti-Mi-2, anti-SRP), immunoprecipitation (anti-TIF1γ, anti-NXP2, anti-SAE and anti-MDA5 and anti-Jo-1, anti-PL-7, anti-PL-12, anti-Mi-2, anti-SRP confirmation) | | Anti-Jo-1: 62/337 (18.4%)  Anti-PL7: 4/337 (1.2%) Anti-PL-12: 1/337 (0.3%)  Anti-Mi-2: 26/337 (7.7%)  Anti-SRP: 14/337 (4.2%)  Anti-TIF1-γ: 12/337 (3.6%)  Anti-SAE: 4/337 (1.2%)  Anti-NXP-2: 4/337(1.2%) | |
| 78 | Paradowska 2016 | It does not report autoantibodies prevalences | | | | | | | |
| 79 | Uruha 2015 | It does not report autoantibodies prevalences | | | | | | | |
| 80 | Limaye 2016 | To determine the prevalence and serological associations of anti-cN1A in South Australian patients with a  definitive histological diagnosis of IBM, made by accepted histological criteria | 58 IBM patients | | Adelaide, South Australia, Australia | ELISA | | cN1A: 24/69 (34.8%)  Anti-Ro52: 6/56 (10.7%) Anti-PMScl100: 1/56 (1.8%) Anti-PL7: 1/56 (1.8%) | |
| 81 | Ali 2016 | It does not address Idiopathic Inflammatory Myopathies | | | | | | | |
| 82 | Lopez de Padilla 2015 | It does not report autoantibodies prevalences | | | | | | | |
| 83 | Taniakou 2016 | Review | | | | | | | |
| 84 | Moghadam 2016 | To determine the association of anti–MDA-5 autoantibody with ILD, RPILD, and survival in US patients with CADM and classic DM. | 122 DM/ADM patients  61 DM patients  61 ADM patients | | Pittsburg, Pennsylvania, USA | ELISA by MBL | | Anti-MDA-5: 8/61 (13.1%) Anti-MDA-5: 8/61 (13.1%)  Anti-MDA-5: 16/ 122 (13.1%) | |
| 85 | Wodkowski 2015 | It does not address Idiopathic Inflammatory Myopathies | | | | | | | |
| 86 | Rojas 2015 | It does not address Idiopathic Inflammatory Myopathies | | | | | | | |
| 87 | Ascherman 2015 | Review | | | | | | | |
| 88 | Kishi 2015 | Case study | | | | | | | |
| 89 | Muro 2015 | Letters to the editor | | | | | | | |
| 90 | Klein 2015 | To analyze the temporal trend of IMNM incidence in our center over the past 10 years and to explore the role of statins as possible causative agents | 217 IIM patients | | Prague, Hlavni mesto Praha, Czech Republic | ELISA (anti-HMGCR), line-blot by Imtec Human and EUROIMMUNE (anti-Jo-1, anti-Mi-2, anti-Ku, anti-PM-Scl, anti-PM-Scl75, anti-PM-Scl100, anti-PL7, anti-PL12, anti-EJ, anti-OJ, anti-SRP, anti-SSA/Ro, anti-Ro52, anti-SSB/La, anti-Scl-70, U1-RNP) immunoprecipitation (anti-TIF-1γ, anti-MDA5, anti-NXP2, anti-Zo, anti-EIF3, anti-RNAP-I, anti-RNAP-II, anti-RNAP III) | | Anti-HMGCR: 15/217 (6.9%)  Anti-SRP: 4/217 (1.9%)  Anti-Jo-1: 2/217 (0.9%) Anti-Ku: 1/217 (0.5%)  Anti-Ro52: 1/217 (0.5%) | |
| 91 | Colanfrancesco 2015 | Paper was not accesed | | | | | | | |
| 92 | Vencovský 2015 | Letter to the editor | | | | | | | |
| 93 | Jearn 2015 | Letter to the editor | | | | | | | |
| 94 | Lega 2015 | It does not report autoantibodies prevalences | | | | | | | |
| 95 | Chen 2015 | To comprehensively assess the profiles of MSAs in Chinese patients with PM/DM and compare them with a Japanese cohort. | Chinese patients:  145 PM/DM  68 DM  52 PM  Japanese patients:  165 PM/DM  56 DM  57 PM | | China and Japan | ELISA (anti-MDA5), immunoprecipitation (anti-ARS, anti-SRP, anti-Ku), immunoprecipitation-immunnoblotting (anti-TIF1γ, anti-Mi-2, anti-SAE, anti-NXP2) | | Chinese patients:  PM/DM  Anti-ARS: 40/145 (27.6%)  Anti-MDA5: 53/145 (36.6%)  Anti-NXP2: 7/145 (4.8%)  Anti-TIF1γ: 8/145 (5.5%)  Anti-SRP: 2/145 (1.4%)  Anti-Mi-2: 6/145 (4.1%)  Anti-Ku: 1/145 (0.7%)  Anti-SAE: 1/145 (0.7%)  DM  Anti-ARS: 10/68 (14.7%)  Anti-MDA5: 31/68 (45.6%)  Anti-NXP2: 6/68 (8.8%)  Anti-TIF1γ: 8/68 (11.8%)  Anti-SRP: 0/68 (0%)  Anti-Mi-2: 5/68 (7.4%)  Anti-Ku: 0/68 (0%)  Anti-SAE: 1/68 (1.5%)  PM  Anti-ARS: 28/52 (53.8%)  Anti-MDA5: 0/52 (0%)  Anti-NXP2: 1/52 (1.9%)  Anti-TIF1γ: 0/52 (0%)  Anti-SRP: 2/52 (3.8%)  Anti-Mi-2: 0/52 (0%)  Anti-Ku: 1/52 (1.2%)  Anti-SAE: 0/52 (0%)  Japanese patients:  PM/DM  Anti-ARS: 66/165 (40%)  Anti-MDA5: 26/165 (15.8%)  Anti-NXP2: 6/165 (3.6%)  Anti-TIF1γ: 14/165 (8.5%)  Anti- SRP: 13/165 (7.9%)  Anti-Mi-2: 4/165 (2.4%)  Anti-Ku: 1/165 (0.6%)  Anti-SAE: 1/165 (0.6%)  DM  Anti-ARS: 26/56 (46.4%)  Anti-MDA5: 3/56 (5.4%)  Anti-NXP2: 5/56 (8.9%)  Anti-TIF1γ: 12/56 (21.4%)  Anti-SRP: 1/56 (1.8%)  Anti-Mi-2: 4/56 (7.1%)  Anti-Ku: 0/56 (0%)  Anti-SAE: 1/56 (1.8%)  PM  Anti-ARS: 25/57 (43.9%)  Anti-MDA5: 0/57 (0%)  Anti-NXP2: 0/57 (0%)  Anti-TIF1γ: 0/57 (0%)  Anti-SRP: 12/57 (21.1%)  Anti-Mi-2: 0/57 (0%)  Anti-Ku: 1/57 (1.8%)  Anti-SAE: 0/57 (0%) | |
| 96 | Muro 2015 | To screen these antibodies in 600 patients with various autoimmune conditions from a single center in Japan and we investigated their clinical significance in Japanese patients | 133 patients with DM/PM | | Nagoya-Shi, Aichi, Japan | ELISA, immunoprecipitation | | PM Scl-100: 3/133 (3.99%)  PM Scl-75. 3/133 (3.99%) | |
| 97 | Goyal 2015 | To explore phenotypic differences between individuals with IBM who are seropositive for the NT5c1A antibody compared with those who are seronegative | 25 patients with IBM | | Missouri, Texas, USA | ELISA, Western blot | | Anti-NT5c1A: 18/25 (72%) | |
| 98 | Fujimoto 2015 | It does not report autoantibodies prevalences | | | | | | | |
| 99 | Hozumi 2015 | To evaluate the clinical significance of anti-ARS antibodies in PM/DM-ILD patients. | 48 PM/DM-ILD patients | | Kyoto, Kyoto, Japan | ELISA by MESACUP (anti-Jo-1, anti-PL7, anti-PL12, anti-EJ, anti-KS), RNA-immunoprecipitation (anti-Jo-1, anti-PL7, anti-PL12, anti-EJ, anti-KS) | | Anti-ARS: 23/48 (48%)  Anti-PL7: 8/48 (16.7%)  Anti-Jo-1: 6/48 (12.5%) Anti-PL12: 4/48 (8.3%) Anti-KS: 3/48 (6.2%)  Anti-EJ: 3/48 (6.2%) | |
| 100 | Watanabe 2015 | To elucidate the clinical significance of anti-HMGCR antibodies in Japanese patients with inflammatory myopathies or MG. | 75 patients with IIM | | Tokyo, Tokyo, Japan | ELISA, immunoprecipitation | | IIM: Anti-HMGCR: 9/75 (12%)  IMNM: Anti-HMGCR: 8/26 (30.8%)  IBM: Anti-HMGCR: 1/24 (4.2%)  PM: Anti-HMGCR: 0/7 (0%)  DM: Anti-HMGCR: 0/18 (0%) | |
| 101 | Lefévre 2015 | It does not address Idiopathic Inflammatory Myopathies | | | | | | | |
| 102 | Narang 2015 | To identify clinical and serologic correlates of cutaneous ulcers in DM | 104 DM patients | | Stanford, California, USA | Not reported | | Anti-MDA5: 16/104 (15.4%) | |
| 103 | Kobayashi 2015 | It does not report autoantibodies prevalences | | | | | | | |
| 104 | Bodoki 2014 | To evaluate the prevalence of these MSAs in IIM and their association with clinical characteristics and the disease course. | 337 patients with IIM | | Debrecen, Debrecen, Hungary | Line-blot by Orgentec Diagnostika (anti-Jo-1, anti-PL7, anti-PL12, anti-Mi-2, anti-SRP), immunoprecipitation (anti-TIF1γ, anti-NXP2, anti-SAE, anti-MDA5) | | Anti- TIF1γ: 12/337 (3.6%)  Anti-NXP2: 4/337 (1.2%)  Anti-SAE: 4/337 (1.2%)  Anti-Jo-1: 62/337 (18.4%)  Anti-Mi2: 26/337 (7.7%)  Anti-SRP: 14/337(4.2%)  Anti PL-12: 1/337 (0.3%) | |
| 105 | Ceribelli 2014 | To examine the clinical significance of anti-MDA5 antibodies in a cohort of European Caucasian patients with PM/DM | 76 PM/DM patients  34 DM patients  30 PM patients | | Milan, Lombardia, Italy | ELISA (anti-MDA5), immunoprecipitation, immunoprecipitation western-blot (anti-MDA5 confirmation) | | DM/PM:  Anti-MJ: 8/76(10.5%)  Anti-MDA5: 5/76 (6.6%)  Anti-Jo1: 5/76 (6.6%)  Anti p155/140: 5/76 (6.6%)  Anti SRP: 3/76 (4%)  Anti EJ: 3/76 (4%)  Anti-Mi-2: 3/76 (4%)  Anti-OJ: 2/76 (2.6%)  Anti PM/Scl: 6/76 (7.9%)  Anti U1RNP: 4/76 (5.3%)  DM:  Anti-MJ: 8/34 (23.5%)  Anti-MDA5: 5/34 (14.7%)  Anti-Jo1: 0/34 (0%)  Anti p155/140: 4/34 (11.8%)  Anti SRP: 0/34 (0%)  Anti EJ: 2/34 (5.9%)  Anti-Mi-2: 2/34 (5.9%)  Anti-OJ: 0/34 (0%)  Anti PM/Scl: 3/34 (8.8%)  Anti U1RNP: 2/34 (5.9%)  PM:  Anti-MJ: 0/30 (0%)  Anti-MDA5: 0/30 (0%)  Anti-Jo1: 5/30 (16.7%)  Anti p155/140: 1/30 (3.3%)  Anti SRP: 3/30 (10%)  Anti EJ: 1/30 (3.3%)  Anti-Mi-2: 1/30 (3.3%)  Anti-OJ: 2/30 (6.7%)  Anti PM/Scl: 3/30 (10%)  Anti U1RNP: 2/30 (6,7%) | |
| 106 | Cuesta 2014 | To determine the association between MSAs and MAAs in ADM Spanish patients | 11 ADM patients | | Madrid, Madrid, España | Immunoprecipitation (anti-p155, anti-MDA5), line-blot by BlueDot Polymyositis/Scleroderma, D-Tek (anti-Jo1, anti-Mi2, anti-SRP, anti-Pm-Scl, anti-Ro52), ELISA by QUANTA LITE, Inova Diagnostic Inc (anti-SSA/Ro, anti-SSB/La, anti-Sm, anti-RNP).  Anti-PL7, anti-PL12, anti-SRP, anti-TIF1γ, anti-Ku method detection no reported. | | Anti-Jo-1: 0/11 (0%) Anti-PL7: 0/11 (0%)  Anti-PL12: 0/11 (0%)  Anti-Mi-2: 0/11 (0%)  Anti-SRP: 0/11 (0%)  Anti-TIF-1γ: 4/11 (36.4%)  Anti-MDA5: 3/11 (27.3%)  Anti-U3-RNP: 0/11 (0%)  Anti-Ku: 0/11 (0%)  Anti-PM-Scl: 0/11 (0%) | |
| 107 | Aguila 2014 | It does not address Idiopathic Inflammatory Myopathies | | | | | | | |
| 108 | Di Rollo 2014 | Paper was not accesed | | | | | | | |
| 109 | Morgan 2014 | It does not address Idiopathic Inflammatory Myopathies | | | | | | | |
| 110 | Klein 2014 | To determine the prevalence, distribution, and clinical manifestations of arthritis in a cohort of patients with IIM | 106 IIM patients | | Prague, Hlavni mesto Praha, Czech Republic | Line-blot by mtec Human, and Western blot-myositis by EUROIMMUNE (anti-Jo-1, anti-Mi-2, anti-Ku, anti-PM-Scl, anti-PM-Scl75, anti-PM-Scl100, anti-PL-7, anti-PL-12, anti-EJ, anti-OJ, anti-SRP, anti-Ro, anti-Ro52, anti-SSB/La, anti-Scl-70, anti-U1-RNP).  Immunoprecipitation (anti-TIF1γ, anti-MDA5, anti-NXP2, anti-Zo, anti-EIF, anti-RNAP-I, anti-RNAP-II, anti-RNAP-III | | Anti-Jo1: 29/106 (27.4%)  Anti-Ro52: 35/106 (32.7%)  Anti-Ro: 12/106 (11.5%)  Anti-PM-Scl: 13/106 (12.3%)  Anti-Mi-2: 7/106 (6.7%)  Anti-TIF-1γ: 9/106(8.2%) | |
| 111 | Labrador 2014 | To evaluate the prevalence and clinical  manifestations of anti-MDA5-positive DM patients from a single center in Barcelona | 117 DM, and 15 ADM | | Barcelona, Catalonia, Spain | ELISA (anti-MDA5, anti-TIF1g), immunoblot (anti-MDA5), immunoprecipitation (anti-Mi-2, anti-SRP, anti-Ro52, anti-Ro60, anti-La, anti-PM/Scl, anti-p155, and anti-U1RNP) | | DM/CADM  Anti-MDA5: 14/117 (12%)  DM:  Anti-MDA5: 6/102 (5.7%)  CADM:  Anti-MDA5: 8/15 (53.3%) | |
| 112 | Zou 2014 | It does not report autoantibodies prevalences | | | | | | | |
| 113 | Lega 2014 | Meta-analysis, it does not report autoantibodies prevalences per city | | | | | | | |
| 114 | Dubey 2014 | It does not address Idiopathic Inflammatory Myopathies | | | | | | | |
| 115 | Mauhin 2014 | It does not report autoantibodies prevalences | | | | | | | |
| 116 | Wang 2014 | To describe the clinical and  histopathological features of 16 Chinese patients with anti-SRP antibodies. | 123 patients with IIM  16 PM patients  42 DM patients  3 IBM patients  18 non-specific patients  44 IMNM patients | | Beijing, Beijing, China | Line-blot by EUROIMMUNE | | IIM:  Anti-SRP: 16/123 (13.0%)  Anti-Ro52: 6/123 (4.9%)  Anti-PM-Scl75: 1/123 (0.8%)  PM:  Anti-SRP: 0/16 (10%)  Anti-Ro52: 0/16 (0%)  Anti-PM-Scl75: 0/16 (0%)  DM:  Anti-SRP: 0/42 (0%)  Anti-Ro52: 0/42 (0%)  Anti-PM-Scl75: 0/42 (0%)  IBM:  Anti-SRP: 0/3 (0%)  Anti-Ro52: 0/3 (0%)  Anti-PM-Scl75: 0/3 (0%)  IMNM:  Anti-SRP: 16/44 (36.4%)  Anti-Ro52: 6/44 (13.7%)  Anti-PM-Scl75: 1/44 (2.3%) | |
| 117 | Taborda 2014 | To analyze the mortality and to review the main factors contributing to it in IIM patients. | 90 patients with myositis | | London, London, UK | Medical records | | Anti-Jo1:18/ 90 (20%)  Anti-SRP: 3/90 (3.3%)  Anti-EJ: 1/90 (1.11%)  Anti-Mi-2: 3/90 (3.3%) | |
| 118 | Neri 2014 | To retrospectively assess the prevalence  and clinical characteristics of cancer-associated IIM | 162 PM/DM patients | | Pisa, Toscana, Italy | Not reported | | Anti-Jo-1: 6/162 (3.7%)  Anti-Ro/SSA: 6/162 (3.7%)  Anti-La/SSB: 2/162 (1.2%)  Anti-RNP 1/162 (0.6%) | |
| 119 | Teruya 2013 | It does not report autoantibodies prevalences | | | | | | | |
| 120 | Tosounidou 2013 | Letter to the editor | | | | | | | |
| 121 | Fiorentino 2013 | To develop sensitive, specific assays to detect antibodies against  TIF-1γ and nuclear matrix protein NXP-2 and to evaluate their association with malignancy in DM. | DM patients from the Stanford University Dermatology Clinic  (n = 111) and the Johns Hopkins Myositis Center (n =  102) | | Stanford, California, USA and Baltimore, Maryland, USA | ELISA (Anti–Jo-1, anti–Ro52), Immunoprecipitation (Anti-TIF1γ, anti-NXP2, anti-Mi-2, anti-SAE1/2) | | Stanford:  Anti-NXP-2: 15/111 (13.5%)  Anti-TIF-1g: 42/111 (37.8%)  Baltimore:  Anti-NXP-2: 22/102 (21.6%)  Anti-TIF-1g: 40/102 (39.2%) | |
| 122 | Cavanazza 2013 | Paper was not accesed | | | | | | | |
| 123 | Shah 2013 | Sample size per city was small | | | | | | | |
| 124 | Hervier 2013 | It does not report autoantibodies prevalences | | | | | | | |
| 125 | Hamaguchi 2013 | It does not report autoantibodies prevalences | | | | | | | |
| 126 | Kozu 2013 | Paper was not accesed | | | | | | | |
| 127 | Petri 2013 | To determine the prevalence and clinical association of MSAs in two Mexican cohorts with PM/DM, focusing on anti-Mi-2 autoantibodies. | 61 DM patients | | Guadalajara, Jalisco, Mexico and Mexico City, Mexico, Mexico | Immunoprecipitation, ELISA (anti-Ro52 and anti-Jo-1) | | Guadalajara:  Anti-Jo-1: 0/17 (0%)  Anti-Mi-2: 2/17 (12%)  Anti-p155/140: 6/17 (35%)  Anti-MJ/NXP2: 1/17 (6%)  Anti-SAE: 0/17 (0%)  Anti-SRP: 1/17 (6%)  Anti-PMScl: 2/17 (12%)  Anti-U1RNP: 0 /17 (0%)  Anti-Ro60: 4/17 (24%)  Anti-Ro52: 5/17 (29%)  Mexico city:  Anti-Jo-1: 2/44 (4%)  Anti-Mi-2: 26/44 (59%)  Anti-p155/140: 4/44 (9%)  Anti-MJ/NXP2: 2/44 (4%)  Anti-SAE: 2/44 (4%)  Anti-SRP: 0/44 (0%)  Anti-PMScl: 1/44 (2%)  Anti-U1RNP: 1/44 (2%)  Anti-Ro60: 5/44 (11%)  Anti-Ro52: 9/44 (21%) | |
| 128 | Cavagna 2013 | Letter to the editor | | | | | | | |
| 129 | Ishigaki 2013 | To determine whether skin ulcer can be used as a predictive and prognostic factor of acute/subacute IL in Japanese patients with DM | 39 DM patients | | Honkomagome, Tokyo, Japan | ELISA (anti-Jo-1), immunoprecipitation (anti-MDA5/CADM-140) | | Anti-Jo-1: 10/39 (25.7%) | |
| 130 | Marie 2013 | It does not report autoantibodies prevalences | | | | | | | |
| 131 | Shah 2013 | To better define the major clinical subgroup phenotypes of JIIM. | 387 JDM/JPM patients  354 JDM patients  33 JPM patients | | Bethesda, Maryland, USA | RNA and protein immunoprecipitation, doble immunodifussion, IP-blotting (anti-p155/140 and anti-MJ/NXP2 confirmation). | | JDM/JPM  Anti-p155/140: 123/387 (31.8%)  Anti-MJ/NXP-2: 79/387 (20.4%)  Anti-ARS: 13/387 (3.4%)  Anti-Jo-1: 8/387 (2.1%)  Anti-PL12: 3/387 (0.8%)  Anti-EJ: 1/387 (0.3%)  Anti-KS: 1/387 (0.3%)  Anti-Mi-2: 10/387 (2.6%)  Anti-SRP: 6/387 (1.6%)  Anti-Ro: 21/387 (5.4%)  Anti-U1RNP: 11/387 (2.8%)  Anti-PM-Scl: 11/387 (2.8%)  Anti-Sm: 1/387 (0.3%)  Anti-La: 0/387 (0%)  Anti-Ku: 1/387 (0.3%)  Anti-SAE/SUMO: 1/387 (0.3%)  JDM  Anti-p155/140: 123/354 (34.7%)  Anti-MJ/NXP-2: 76/354 (21.5%)  Anti-ARS: 10/354 (2.8%)  Anti-Jo-1: 5/354 (1.4%)  Anti-PL12: 3/354 (0.8%)  Anti-EJ: 1/354 (0.3%)  Anti-KS: 1/354 (0.3%)  Anti-Mi-2: 10/354 (2.8%)  Anti-SRP: 0/354 (0%)  Anti-Ro: 19/354 (5.4%)  Anti-U1RNP: 7/354 (2%)  Anti-PM-Scl: 9/354 (2.6%)  Anti-Sm: 0/354 (0%)  Anti-La: 0/354 (0%)  Anti-Ku: 1/354 (0.3%)  Anti-SAE/SUMO: 1/354 (0.3%)  JPM  Anti-p155/140: 0/33 (0%)  Anti-MJ/NXP-2: 3/33 (9.1%)  Anti-ARS: 3/33 (9.1%)  Anti-Jo-1: 3/33 (9.1%)  Anti-PL12: 0/33 (0%)  Anti-EJ: 0/33 (0%)  Anti-KS: 0/33 (0%)  Anti-Mi-2: 0/33 (0%)  Anti-SRP: 6/33 (18.2%)  Anti-Ro: 2/33 (6.1%)  Anti-U1RNP: 4/33 (12.1%)  Anti-PM-Scl: 2/33 (6.1%)  Anti-Sm: 1/33 (3%)  Anti-La: 0/33 (0%)  Anti-Ku: 0/33 (0%)  Anti-SAE/SUMO: 0/33 (0%) | |
| 132 | Muro 2013 | To study the importance of antiMDA5ab levels and of ferritin and IL-18 concentrations as predictors for the prognosis of ILD/DM in this study | 95 DM patients | | Nagoya-Shi, Aichi, Japan | ELISA | | Anti-MDA5: 25/95 (26.3%) | |
| 133 | Da Silva 2012 | Letter to the editor | | | | | | | |
| 134 | Marie 2013 | To assess the outcome of ILD in anti–Jo-1 patients with antisynthetase syndrome and to determine predictive variables | 346 PM/DM c patients | | Rouen, Houte-Normandie, France | Immunodiffusion and confirmation by ELISA | | Anti-Jo-1: 91/346 (26.3%) | |
| 135 | Salvador 2012 | It does not report autoantibodies prevalences | | | | | | | |
| 136 | Gono 2012 | To investigate the HLA–DRB1 genotype in patients with anti–MDA-5 antibody | 33 PM/DM patients | | Tokyo, Tokyo, Japan | Immunoprecipitation (anti-MDA5-140), ELISA (anti-MDA5/CADM-140), RNA-immunoprecipitation (anti-Jo-1, anti-EJ, anti-OJ, anti-PL7, anti-PL12, anti-SRP, anti-Ku, anti-U1RNP) | | Anti–Jo-1: 16/33 (48.5%)  Anti-EJ: 10/33 (30.3%)  Anti–PL-7: 4/33 (12.1%)  Anti–PL-12: 3/33 (9.1%)  Anti-OJ: 0/33 (0%) | |
| 137 | Tarricone 2012 | To set up an unlabelled protein immunoprecipitation technique for MSA identification in the sera of myositis patients | 130 DM/PM patients  75 DM patients  43 PM patients | | Padova, Veneto, Italy | Immunoblotting and RNA immunoprecipitation | | Anti-SAE: 5/118 (4.2%)  Anti-Jo-1= 11/118 (9.3%)  Anti-PL-12= 6/118 (5.1%)  Anti-PL-7=4/118 (3.4%)  Anti-OJ= 1/118 (0.8%)  Anti-SRP= 5/118 (4.2%)  Anti-Ro/SSA= 10/118 (8.5%)  Anti-U1RNP = 4/118 (3.4%)  Anti-La/SSB = 2/118 (1.7%) | |
| 138 | Szodoray 2012 | It does not report autoantibodies prevalences | | | | | | | |
| 139 | Fujimoto 2012l | To detect anti-SAE in a Japanese DM cohort and to assess its clinical correlations. | 456 DM japanese patients | | Kanazawa, Ishikawa, Japan | Immunoprecipitation, western-blotting (anti-SAE confirmation) | | Anti-SAE: 7/456 (1.5%)  Anti -Ro/SS-A: 2/456 (0.4%) | |
| 140 | Yosipovitch 2012 | It does not report autoantibodies prevalences | | | | | | | |
| 141 | Riverio 2012 | Case Report | | | | | | | |
| 142 | Hervier 2012 | Multicentric study, it does not report autoantibodies prevalences per city | | | | | | | |
| 143 | Chuan 2012 | To identify associated factors with an increased risk of developing ILD in IIM patients | 107 IIM patients | | London, Greater London, UK | Dot-blot, indirect immunofluorescence, ELISA | | Anti-Jo-1: 20/107 (18.6%) | |
| 144 | Labirua 2012 | Multicentric study, it does not report autoantibodies prevalences per city | | | | | | | |
| 145 | Chaisson 2012 | Case reports | | | | | | | |
| 146 | Gono 2012 | To investigate the precise clinical characteristics and to analyse the association between anti-MDA5 and disease status in patients with anti-MDA5ab positive DM | 142 DM patients    53 ADM patients | | Tokyo, Tokyo, Japon | ELISA | | DM  Anti-MDA5: 5/142 (3.5%)  ADM  Anti-MDA5: 22/53 (41.5%)  DM + ADM  Anti-MDA5: 27/195 (13.9%) | |
| 147 | Ceribelli 2012 | To analyze the prevalence and clinical significance of anti-MJ antibodies in a cohort of adult Italian PM/DM patients | 58 PM/DM patients | | Brescia, Lombardia, Italy | Immunoprecipitation, ELISA (anti-MJ/NXP-2, anti-Jo-1), Western blot-immunoprecipitation (anti-MJ/NXP-2 confirmation) | | Anti-MJ: 10/58 (17.2%)  Anti-Jo-1: 6/58 (10.3%)  Anti-p155/140: 3/58 (5.2%)  Anti-SRP: 3/58 (5.2%)  Anti-EJ: 2/58 (3.4%)  Anti-Mi-2: 1/58 (1.7%)  Anti-OJ: 1/58 (1.7%)  Anti-PM/Scl: 6/58 (10.3%)  Anti-Ro: 2/58 (3.4%)  Anti-Su: 2/58 (3.4%) | |
| 148 | Koga 2012 | To investigate the clinical value of anti-MDA5 Ab for DM patients in a single cohort | 79 DM patients, | | Nagasaki-Shi, Aichi, Japan | Immunoprecipitation (anti-MDA5/CADM-140, anti-p155/140, anti-ARS), ELISA (anti-MDA5) | | Anti-MDA5: 17/79 (21.5%)  Anti-p140: 16/79 (20.2%)  Anti-p155/140: 7/79 (8.9%)  Anti-ARS: 30/79 (37.9) | |
| 149 | Bronze 2012 | It does not address Idiopathic Inflammatory Myopathies | | | | | | | |
| 150 | Mimori 2012 | It does not report autoantibodies prevalences | | | | | | | |
| 151 | Ellis 2012 | To determine the clinical features and comorbidities associated with Necrotizing Myopathy and to compare these with patients with histologically determined IIM. | 64 IMNM patients | | Adelaide, South Australia, Australia | Lineblot by EUROIMMUNE | | Anti-SRP: 0/23 (0%)  Anti-Ro52: 2/23 (8.7%)  Anti-PMScl75: 5/23 (21.7%)  Anti-Ku: 1/23 (4.3%) | |
| 152 | Lakota 2012 | It does not report autoantibodies prevalences | | | | | | | |
| 153 | Polimeni 2012 | It does not address Idiopathic Inflammatory Myopathies | | | | | | | |
| 154 | Muro 2011 | To assess the growing prevalence of ADM and the geographical incidence of anti-MDA-5-positive patients | 95 DM patients | | Nagoya-Shi, Aichi, Japan | Immunoprecipitation | | DM + CADM:  Anti-MDA5: 26/95 (27.4%) | |
| 155 | Chinoy 2011 | This study examined  whether smoking was associated with the development of anti-Jo-1 antibodies in HLA-DRB1*03-positive IIM | 557 IIM patients | | Budapest, Budapest, Hungary  Prague, Hlavni mesto Praha, Czech Republic  Stockholm, Stockholms Land, Sweden  London, Greater London, United Kingdom | Line-blot by EUROIMMUNE, immunoprecipitation (anti-Jo-1 UK samples) | | Anti-Jo-1 overall: 115/557 (20.6%)  Anti-Jo-1 Hungary: 29/181 (16%)  Anti-Jo-1 Czech Republic: 50/183 (27.3%)  Anti-Jo-1 Sweden: 14/94 (15%)  Anti-Jo-1 UK: 22/99 (22,2%) | |
| 156 | Castañeda 2011 | Letter to the editor | | | | | | | |
| 157 | Ikeda 2011 | To evaluate clinical characteristics, especially skin manifestations, in association with DM associated autoantibodies | 55 DM patients | | Tokyo, Tokyo, Japan | Immunoprecipitation | | Anti-CADM/MDA5: 6/55 (10.9%)  Anti-155/140: 9/55 (16.3%)  AntI-ARS: 8/55 (14.5%)  Anti-Mi-2: 6/55 (10.9%)  Anti-Jo-1: 3/55 (4.5%)  Anti-PL-7: 2/55 (3.6%)  Anti-PL12: 3/55 (4.5%) | |
| 158 | Pakpzdi 2011 | It does not address Idiopathic Inflammatory Myopathies | | | | | | | |
| 159 | Dubey 2011 | It does not address Idiopathic Inflammatory Myopathies | | | | | | | |
| 160 | Andres 2011 | Letter to the editor | | | | | | | |
| 161 | Bader 2011 | It does not report autoantibodies prevalences | | | | | | | |
| 162 | Mammen 2011 | Review | | | | | | | |
| 163 | Satoh 2011 | It does not report autoantibodies prevalences | | | | | | | |
| 164 | Fiorentino 2011 | To understand the role of MDA5 in DM skin inflammation by testing it to determine if a specific cutaneous phenotype is associated with MDA5 reactivity. | 77 patients with DM | | Stanford, California | Immunoprecipitation (anti-MDA5/CADM-140, anti-Mi-2, anti-Ro60), ELISA (anti-Jo-1, anti-Ro-52) | | Anti-Mi-2: 9/77 (11.6%)  Anti-MDA5/CADM-140: 10/77 (13%)  Anti-Jo-1: 4/77 (5.2%)  Anti-Ro52: 15/77 (19.5%)  Anti-Ro60 5/77 (6.5%) | |
| 165 | Nagashima 2011 | Letter to the editor | | | | | | | |
| 166 | Watanabe 2011 | It does not address Idiopathic Inflammatory Myopathies | | | | | | | |
| 167 | Hamaguchi 2011 | To clarify the association of clinical and  prognostic features with dermatomyositis specific autoantibodies in Japanese patients with DM. | 410 DM/PM patients  376 DM patients  34 PM patients | | Kanazawa, Aomori, Japan | Immunoprecipitation, Inmunoblot (anti-MDA5/CADM-140 confirmation), ELISA (anti-MDA5/CADM-140 confirmation) | | DM/PM  Anti–Jo-1: 25/410 (6.1%)  Anti-ARS: 55/410 (13.4%)  Anti-SRP: 9/410 (2.2%)  Anti–Mi-2: 9/410 (2.2%)  Anti–155/140: 25/410 (6.1%)  Anti–CADM-140: 43/410 (10.5%)  Anti-U1RNP: 12/410 (2.9%)  Anti-PM-Scl: 0/410 (0%)  Anti-Ku: 5/410 (1,2%)  DM  Anti–Jo-1: 21/376 (5.6%)  Anti-ARS: 49/376 (13%)  Anti-SRP: 7/376 (1.9%)  Anti–Mi-2: 9/376 (2.4%)  Anti–155/140: 25/376 (6.6%)  Anti–CADM-140: 43/376 (11.4%)  Anti-U1RNP: 11/376 (2.9%)  Anti-PM-Scl: 0/376 (0%)  Anti-Ku: 2/376 (0.5%)  PM  Anti–Jo-1: 4/34 (11.8%)  Anti-ARS: 6/34 (17.6%)  Anti-SRP: 2/34 (5.8%)  Anti–Mi-2: 0/34 (0%)  Anti–155/140: 0/34 (0%)  Anti–CADM-140: 0/34 (0%)  Anti-U1RNP: 1/34 (2.9%)  Anti-PM-Scl: 0/34 (0%)  Anti-Ku: 3/34 (8.8%) | |
| 168 | Nakajima 2011 | To evaluate the frequency of autoantibodies and the accompanying clinical features in patients with IIM overlapped to Rheumatoid Arthritis (IIM-RA) and in patients with IIM without RA. | 142 DM/PM patients | | Tokyo, Tokyo, Japan | Double Immunodifussion (anti-Jo-1, anti-SSA/Ro, anti-U1RNP), Immunoprecipitation (anti-Jo-1 confirmation) | | Anti-Jo-1: 26/130 (20.2 %) | |
| 169 | Kanetaka 2011 | Clinical report | | | | | | | |
| 170 | Mammen 2011 | To inducing a self-limited myopathy, statin use is associated with an  immune-mediated necrotizing myopathy with autoantibodies recognizing ~ 200 and ~100 kDa autoantigens | 750 IIM patients | | Baltimore, Maryland, USA | ELISA, immunoprecipitation | | Anti-HMGCR: 45/750 (6%) | |
| 171 | Kang 2010 | To investigate the association between myositis autoantibodies and clinical subsets of inflammatory myositis in Korean patients | 49 PM/DM patietns  11 PM patients  38 DM patients | | Seoul, South Korea | Immunoprecipitation | | Anti-p140: 9/49 (18.4%)  Anti-p155/140: 8/49(16.3%)  Anti-Mi-2: 7/49 (14.3%)  Anti-ARS: 6/49 (12.2%)  Anti-SRP: 1/49 (2.0%) | |
| 172 | Yanaba 2011 | It does not address Idiopathic Inflammatory Myopathies | | | | | | | |
| 173 | Ranque 2010 | It does not address Idiopathic Inflammatory Myopathies | | | | | | | |
| 174 | Ghirardello 2010 | To evaluate the accuracy of a commercial line blot assay for myositis diagnosis. | 208 IIM patients | | Padova, Veneto, Italy | Line-blot by EUROIMMUNE (anti-Jo-1, anti-PL7, anti-PL12, anti-PMScl, anti-Mi-2, anti-Ku, anti-Ro52) immunoprecipitation-immunoblotting (confirmation) | | Anti-Jo-1: 43/208 (20.1%)  Anti-Mi-2: 9/208 (4.3%)  Anti-PM/Scl: 9/208 (4.3%)  Anti-Ku: 10/208 (4.8%)  Anti-Ro52: 49/208 (23.6%) | |
| 175 | Marie 2010 | To assess the long-term outcome of esophageal complications in the group of patients receiving intravenous immunoglobulins (IVIG) | 73 patients (39 with PM, 34 with DM) | | Paris, Ile-de-France, France | Medical records | | Anti-Jo-1: 6/73 (8.2%)  Anti-PL7: 2/73 (2.7%)  Anti-PL12: 1/73 (1.4%)  Anti-SRP: 2/73 (2.7%) | |
| 176 | Segura 2010 | It does not address Idiopathic Inflammatory Myopathies | | | | | | | |
| 177 | Ramos 2010 | It does not report autoantibodies prevalences | | | | | | | |
| 178 | Fauchais 2010 | It does not address Idiopathic Inflammatory Myopathies | | | | | | | |
| 179 | Irie 2010 | It does not report autoantibodies prevalences | | | | | | | |
| 180 | Váncsa 2010 | To determine the prevalence of different MAAs and MSA, as well as their association with clinical characteristics, disease course and response to therapy. | 130 PM/DM Hungarian patients | | Debrecen, Debrecen, Hungary | ELISA (Anti-dsDNA, anti-PMScl-70, anti-Jo-1, anti-SSA/Ro, anti-SSB/La), Line-blot by EUROIMMUNE (anti-Jo-1 confirmation, anti-PL7, anti-PL12, anti-SRP, anti-Ku, anti-PMScl-70, anti-U1RNP)  MI-2 method detection no reported | | Anti-Jo-1: 24/130 (18.5%)  Anti-PL7: 2/130 (1.5%)  Anti-Mi-2: 10/130 (7.7%)  Anti-SSA/Ro: 11/130 (8.5%)  Anti-SSB/La: 7/130(5.4%)  Anti-U1RNP: 3/130 (2.3%)  Anti-Ku: 5/130 (3.8%) | |
| 181 | Noda 2010 | Case Report | | | | | | | |
| 182 | Dugar 2010 | It does not report autoantibodies prevalences | | | | | | | |
| 183 | Espada 2009 | To investigate the autoantibody pattern in a pediatric Argentine Caucasian cohort in order to characterize novel autoantibodies | 47 JDM/JPM patients  40 JDM patients  7 JPM patients | | Buenos Aires, Buenos Aires, Argentina | Immunoprecipitation, IP-western blot (anti-MJ/NXP2 confirmation) | | JDM/JPM  Anti-Mi-2: 4/47 (8.5%)  Anti-PMScl: 1/47 (2.1%)  Anti-U1RNP: 3/47 (6.4%)  Anti-Ku: 0/47 (0%)  Anti-MJ: 15/47 (31.9%)  Anti-p155/140: 11/47 (23.4%)  JDM  Anti-Mi-2: 4/40 (10%)  Anti-PMScl: 1/40 (2.5%)  Anti-U1RNP: 3/40 (7.5%)  Anti-Ku: 0/40 (0%)  Anti-MJ: 13/40 (32.5%)  Anti-p155/140: 11/40 (27.5%)  JPM  Anti-Mi-2: 0/7 (0%)  Anti-PMScl: 0/7 (0%)  Anti-U1RNP: 0/7 (0%)  Anti-Ku: 0/7 (0%)  Anti-MJ: 2/7 (28.6%)  Anti-p155/140: 0/7 (0%) | |
| 184 | O’Hanlon 2009 | Review | | | | | | | |
| 185 | Love 2009 | Sample size per city was small | | | | | | | |
| 186 | Mileti 2009 | Concise Report | | | | | | | |
| 187 | Gunawardena, 2009 | Multicentric study, it does not report autoantibodies prevalences per city | | | | | | | |
| 188 | Fujikawa 2009 | To determine the association of distinct clinical subsets with MSAs in Japanese patients with DM. | 30 DM patients | | Nagasaki-Shi, Nagasaki,  Japan | Immunoprecipitation | | Anti-155/140: 5/30 (16.7%)  Anti-140: 8/30 (26.7%)  Anti-ARS: 7/30 (23%) | |
| 189 | Suzuki 2009 | To investigate the efficacy of IVIG for refractory ILD-PM/DM | 5 patients treated with IVIG for refractory ILD-PM/DM resistant | | Hamamatsu, Shikouka, Japan | Medical records | | Anti-Jo1: 1/5 (20%) | |
| 190 | Dugan 2009 | Review | | | | | | | |
| 191 | Kalluri 2009 | It does not report autoantibodies prevalences | | | | | | | |
| 192 | Selva 2009 | To determine the frequency of Obstructive Sleep Apnea in patients diagnosed with IIM | 16 IIM patients | | Barcelona, Catalonia, Spain | ELISA (Anti-SSA/Ro, anti-SSB/La, anti-RNP, anti-Sm, anti-Jo-1), protein and RNA immunoprecipitation (anti–Mi-2, anti-SRP, anti-Ro52, anti-Ro60, anti-SSB/La, anti-PM/Scl, and anti-RNP) | | Anti-PMScl: 5/16 (31.2%)  Anti- ARS: 3/16 (18.7%) | |
| 193 | Takada 2008 | To elucidate the clinical and histopathological features associated with anti-SRP | 23 PM/DM japanese patients | | Nagoya-Shi, Aichi, Japan | Immunoprecipitation | | Anti-SRP: 23/3500 (0.7%) | |
| 194 | Betteridge 2018 | To establish the frequency of anti-SAE autoantibodies in a United Kingdom myositis cohort | 266 IIM patients | | Manchester, Manchester, United Kingdom | Immunoprecipitation | | Anti-SAE: 11/266 (4.1%) | |
| 195 | Chinoy 2008 | It does not report autoantibodies prevalences | | | | | | | |
| 196 | Rozelle 2018 | Case Report | | | | | | | |
| 197 | Tanaka 2008 | It does not report autoantibodies prevalences | | | | | | | |
| 198 | Pullenayegum 2008 | It does not report autoantibodies prevalences | | | | | | | |
| 199 | Rouster-Stevens 2008 | Case Report | | | | | | | |
| 200 | Manlhiot, 2008 | It does not report autoantibodies prevalences | | | | | | | |
| 201 | Needham 2008 | It does not report autoantibodies prevalences | | | | | | | |
| 202 | Gunawardena 2007 | It does not report autoantibodies prevalences per city | | | | | | | |
| 203 | Morinishi 2007 | It does not report autoantibodies prevalences | | | | | | | |
| 204 | O’Hanlon 2014 | It does not report autoantibodies prevalences per city | | | | | | | |
| 205 | Selva-O’callaghan 2006 | To determine the prevalence of MSAs and MAAs and their clinical and immunogenetic correlations in Mediterranean patients with IIM | 88 IIM patients | | Barcelona, Catalonia, Spain | ELISA (Anti-SSA/Ro, anti-SSB/La, anti-RNP, anti-Sm, anti-Jo-1), protein and RNA immunoprecipitation (anti-ARS, anti–Mi-2, anti-SRP, anti-Ro52, anti-Ro60, anti-SSB/La, anti-PM/Scl, and anti-RNP),  Anti-Ku method detection were not reported | | Anti-ARS: 21/88 (23.9%)  Anti-Jo-1: 14/88 (15.9%)  Anti-Mi-2; 6/88 (6.8%)  Anti-SRP: 0/88 (0%)  Anti-Ro60: 20/88 (22.7%)  Anti-Ro52: 18/88 (20.4%)  Anti-PmScl: 10/88 (11.4%)  Anti-U1RNP: 6/88 (6.8%)  Anti-U5RNP: 2/88 (2.3%)  Anti-Ku: 1/88 (1.1%) | |
| 206 | Constantin 2006 | It does not report autoantibodies prevalences | | | | | | | |
| 207 | Zampieri 2006 | Review | | | | | | | |
| 208 | Yamasaki 2006 | To compare the clinical manifestations in these anti–PL-7 positive patients with those in a subset of anti-Jo-1–positive patients with IIM | 36 IIM patients | | Kawasaki, Kanagawa, Japan | Immunoprecipitation, ELISA (anti-Jo-1 confirmation) | | Anti-Jo-1: 8/36 (22%)  Anti-EJ: 1/36 (3%)  Anti-OJ: 1/36 (3%)  Anti-PL-12: 2/36 (6%)  Anti-PL7: 6/36 (16.7%) | |
| 209 | Scalapino, 2006 | It does not address Idiopathic Inflammatory Myopathies | | | | | | | |
| 210 | Chinoy 2006 | To investigate HLA class II associations in PM and DM and to determine how these associations influence clinical and serological differences. | 225 UK IIM patients  117 PM patients  108 DM patients | | London, Greater London, UK | Immunoprecipitation, immunodiffusion (anti-PM-Scl, anti-Jo-1, anti-U1RNP confirmation) | | PM  Anti-Jo-1: 24/117 (20.5%)  Anti-PL-7: 1/117 (0.8%)  Anti-PL-12: 0/117 (0%)  Anti-EJ: 0/117 (0%)  Anti-OJ: 1/117 (0.8%)  Anti-KS: 1/117 (0.8%)  Anti-Mi-2: 1/117 (0.8%)  Anti-SRP: 5/117 (4.3%)  Anti-U1-RNP: 5/117 (4.3%)  Anti-U3-RNP: 0/117 (0%)  Anti-Ku: 0/117 (0%)  Anti-PM-Scl: 5/117 (4.3%)  DM:  Anti-Jo-1: 22/108 (20.4%)  Anti-PL-7: 0 /108 (0%)  Anti-PL-12: 1/108 (0.9%)  Anti-EJ: 1/108 (0.9%)  Anti-OJ: 1/108 (0.9%)  Anti-KS: 1/108 (0.9%)  Anti-Mi-2: 17/108 (15.7%)  Anti-SRP: 2/108 (1.9%)  Anti-U1-RNP: 8/108 (7.4%)  Anti-U3-RNP: 2/108 (1.9%)  Anti-Ku: 2/108 (1.9%)  Anti-PM-Scl: 6/108 (5.6%)  PM/DM  Anti-Jo-1: 46/225 (20.4%)  Anti-PL-7: 1/225 (0.4%)  Anti-PL-12: 1/225 (0.4%)  Anti-EJ: 1/225 (0.4%)  Anti-OJ: 2/225 (0.9%)  Anti-KS: 2/225 (0.9%)  Anti-Mi-2: 18/225 (8%)  Anti-SRP: 7/225 (3.1%)  Anti-U1-RNP: 13/225 (5.8%)  Anti-U3-RNP: 2/225 (0.9%)  Anti-Ku: 2/225 (0.9%)  Anti-PM-Scl: 11/225 (4.9%) | |
| 211 | Engel 2005 | Review | | | | | | | |
| 212 | Hengstman 2016 | It does not report autoantibodies prevalences | | | | | | | |
| 213 | Bugatti 2005 | Case report | | | | | | | |
| 214 | Rios 2005 | To describe demographic  characteristics, clinical features, and functional outcome of Puerto  Rican patients with IIM | Patients with a diagnosis of IIM (PM/DM); 12 were tested | | San Juan, Puerto Rico, USA | Medical records | | Anti-Jo1: 1/12 (8.3%) | |
| 215 | Komura 2005 | Letter to the editor | | | | | | | |
| 216 | Lundberg 2005 | Review | | | | | | | |
| 217 | Sato 2005 | It does not report autoantibodies prevalences | | | | | | | |
| 218 | Satoh 2005 | It does not report autoantibodies prevalences | | | | | | | |
| 219 | Gergely 2005 | To investigate whether the continuing infection with TTV DNA relates to the clinical and laboratory parameters, disease activity, and severity in patients with IIM | 94 IIM patients | | Budapest, Budapest, Hungary | Not reported | | Anti-Jo-1: 13/94 (13.8%) | |
| 220 | Troyanov 2005 | Review | | | | | | | |
| 221 | Sato 2005 | To identify novel autoantibodies specific for DM, especially those specific for CADM | 103 IIM patients  61 PM patients  27 DM patients  15 ADM patients | | Nagoya-Shi, Aichi, Japan | Immunoprecipitation-immunobloting | | PM  Anti-Jo-1: 10/61 (16.4%)  Anti-ARS: 10/61 (16.4%)  Anti-SRP: 5/61 (8.2%)  Anti-Mi-2: 0/61 (0%)  Anti-SSA/Ro: 3/61 (4.9%)  Anti-U1 RNP: 2/61 (3.3%)  Anti-CADM-140: 0/61 (0%)  DM  Anti -Jo-1: 6/27 (22.2%)  Anti-ARS: 2/27 (7.4%)  Anti-SRP: 0/27 (0%)  Anti-Mi-2: 2/27 (7.4%)  Anti-SSA/Ro: 3/27 (11.1%)  Anti-U1 RNP: 2/27 (7.4%)  Anti-CADM-140: 0/27 (0%)  ADM  Anti -Jo-1: 0/15 (0%)  Anti-ARS: 0/15 (0%)  Anti-SRP: 0/15 (0%)  Anti-Mi-2: 0/15 (0%)  Anti-SSA/Ro: 2/15 (13.3%)  Anti-U1 RNP: 0/15 (0%)  Anti-CADM-140: 8/15 (53.3%)  DM + ADM  Anti -Jo-1: 6/42 (14.3%)  Anti-ARS: 2/42 (4.8%)  Anti-SRP: 0/42 (0%)  Anti-Mi-2: 2/42 (4.8%)  Anti-SSA/Ro: 5/42 (11.9%)  Anti-U1 RNP: 2/42 (4.8%)  Anti-CADM-140: 8/42 (19%)  PM+DM+ADM  Anti-Jo-1: 16/103 (15.5%)  Anti-ARS: 12/103 (11.7%)  Anti-SRP: 5/103 (4.9%)  Anti-Mi-2: 2/103 (11.7%)  Anti-SSA/Ro: 8/103 (7.8%)  Anti-U1 RNP: 4/103 (3.9%)  Anti-CADM-140: 8/103 (7.8%) | |
| 222 | Chapman 2005 | Review | | | | | | | |
| 223 | Kondo 2004 | Paper was not accesed | | | | | | | |
| 224 | Shoenfeld 2005 | A meeting review | | | | | | | |
| 225 | Wanchu 2003 | Paper was not accesed | | | | | | | |
| 226 | Kao 2004 | To determine the long-term outcome and associated clinical, serologic, and pathologic features in a cohort of patients with connective tissue disease (CTD) and the anti–signal recognition particle (anti-SRP) autoantibody. | 263 PM/DM patients  134 PM patients    129 DM patients | | Pittsburgh, Pennsylvania, USA | Protein and RNA immunoprecipitation | | PM/DM  Anti-SRP: 16/263 (6.1%)  PM  Anti-SRP: 16/134 (11.9%)  DM  Anti-SRP: 0/129(0%) | |
| 227 | Kamali 2004 | It does not report autoantibodies prevalences | | | | | | | |
| 228 | Okada 2003 | To determine if geoclimatic factors may influence the nature and frequency of DM/PM, and associated autoantibodies around the world. | DM and PM patients among the myositis populations at referral centers in 15 cities on 4 continents. | | Glasgow, Scotland.  Warsaw, Poland  Aachen, Germany  Bethesda, Maryland, US  Montreal, Quebec, Canada  Seoul. Korea  Santiago, Chile  New Delhi, India  Mexico City, Mexico  Guadalajara, Mexico  Guatemala City, Guatemala | Standard immunodiffusion, indirect immunofluorescence, protein and RNA immunoprecipitation | | Anti-Mi-2 positive DM/PM patients  Glasgow, Scotland: 1/15 (6.7%)  Stockholm, Sweden: --  Warsaw, Poland: 1/27 (3.7%)  Nijmegen, The Netherlands: --  Aachen, Germany: 20/147 (13.6%)  Tokyo, Japan:--  Bethesda, Maryland, US: 10/186 (5.4%)  Montreal, Quebec, Canada: 1/31 (3.2%)  Seoul. Korea: 4/51 (7.8%)  Santiago, Chile: 3/13 (23.1%)  Barcelona, Spain: --  New Delhi, India: 9/47 (19.1%)  Mexico City, Mexico: 13/36 (36.1%)  Guadalajara, Mexico: 5/38 (13.2%)  Guatemala City, Guatemala: 18/30 (60%)  Anti-ARS positive DM/PM patients  Glasgow, Scotland: 2/15 (13.3%)  Stockholm, Sweden: --  Warsaw, Poland: 1/27 (3.7%)  Nijmegen, The Netherlands: --  Aachen, Germany: 59/147 (40.1%)  Tokyo, Japan:--  Bethesda, Maryland, US: 48/186 (25.8%)  Montreal, Quebec, Canada: 1/31 (3.2%)  Seoul. Korea: 8/51 (15.7%)  Santiago, Chile: 3/13 (23.1%)  Barcelona, Spain: --  New Delhi, India: 5/47 (10.6%)  Mexico City, Mexico: 4/36 (11.1%)  Guadalajara, Mexico: 0/38 (0%)  Guatemala City, Guatemala: 1/30 (3.3%)  Anti-SRP positive DM/PM patients  Glasgow, Scotland: 0/15 (0%)  Stockholm, Sweden: --  Warsaw, Poland: 0/27 (0%)  Nijmegen, The Netherlands: --  Aachen, Germany: 0/147 (0%)  Tokyo, Japan:--  Bethesda, Maryland, US: 7/186 (3.4%)  Montreal, Quebec, Canada: 1/31 (3.2%)  Seoul. Korea: 3/51 (5.9%)  Santiago, Chile: 0/13 (0%)  Barcelona, Spain: --  New Delhi, India: 1/47 (2.1%)  Mexico City, Mexico: 1/36 (2.8%)  Guadalajara, Mexico: 0/38 (0%)  Guatemala City, Guatemala: 0/30 (0%) | |
| 229 | Bodolay 2003 | It does not address Idiopathic Inflammatory Myopathies | | | | | | | |
| 230 | Marie 2002 | To assess prevalence, characteristics, and long-term outcome ILD in  PM and DM patients. To determine predictive variables of ILD course in PM/DM. | 156 PM/DM patients  90 PM patients  66 DM patients | | Paris, Ile-de-France, France | Medical records | | Anti–Jo-1: 11/156 (7.1%) | |
| 231 | Shamim 2002 | It does not report autoantibodies prevalences | | | | | | | |
| 232 | Kubo 2002 | To determine the prevalence of 52-kd and 60-kd Ro/SS-A antibodies in Japanese patients with PM/DM | 61 PM/DM patients | | Tokio, Tokyo, Japan | Double immunodiffusion (anti-SSA/Ro, anti-SSB/La), immunoprecipitation (anti-Jo-, anti-EJ , anti-OJ, anti-PL7, anti-PL12, anti-SRP, anti- SSA/Ro, anti- SSB/La, anti-Th/To, anti-U3RNP antibodies.), ELISA by MBL (antI-Ro52, anti-Ro60) | | Anti-Ro/SS-A: 4/61 (6.6%)  Anti-La/SS-B: 1/61 (1.6%) | |
| 233 | Hengstman 2002 | To study the clinical and serological characteristics of IIM in patients | 125 IIM patients | | Nijmegen, Gelderland, Netherlands | Immunoprecipitation (anti-Jo-1, anti-ARS, anti-SRP), immunoblotting (anti-Jo-1), and ELISA (anti-Mi-2, anti-Jo-1, anti-Ro52, anti-Ro60, anti-SSB/La, anti-PM/Scl75, anti-PM/Scl100) | | Anti-Jo-1: 25/125 (20%)  Anti-Mi-2: 7/125 (6%)  Anti-SRP: 5/125 (4%) | |
| 234 | Tormey 2001 | It does not address Idiopathic Inflammatory Myopathies | | | | | | | |
| 235 | Gilbert 2001 | Paper was not accesed | | | | | | | |
| 236 | Huemer 2001 | Paper was not accesed | | | | | | | |
| 237 | Brouwer 2001 | It does not report autoantibodies prevalences per city | | | | | | | |
| 238 | Rozman 2000 | Paper was not accesed | | | | | | | |
| 239 | Mygland 2000 | It does not address Idiopathic Inflammatory Myopathies | | | | | | | |
| 240 | Weinstein 2000 | Paper was not accesed | | | | | | | |
| 241 | Rozman 200 | Letters to the editor | | | | | | | |
| 242 | Kubo 2018 | To investigate the clinical and immunological significance of nailfold videocapillaroscopy abnormalities in patients with IIM | | 70 patients with IIM | Kitakyushu, Fukuoka, Japan | | Immunoprecipitation and ELISA by Diarect, Freiburg (anti-MDA5 and anti-Jo-1) | | Anti-MDA5: 16/70 (22.9%)  Anti-ARS: 26/70 (37.1%)  Anti-TIF1g: 9/70 (12.9%)  Anti-Mi-2: 1/70(1.4%) |
