# Supplementary material for: Geographical Latitude Remains as an Important Factor for the Prevalence of Some Myositis Autoantibodies: A Systematic Review
Source: Front Immunol. 2021 Apr 22;12:672008. doi: 10.3389/fimmu.2021.672008 (PMC8100663; doi:10.3389/fimmu.2021.672008)
Supplement: Supplementary file 3 [file Table_2.docx]

**Supplementary Table 2. Prevalence of MSA and MAA according to continents**

| MSA/MAA  $\bar{\boldsymbol{x}}$(min – max) | Continent | | | | *P^&^* |
| --- | --- | --- | --- | --- | --- |
|  | Europe | Asia | America | Oceania |  |
| Anti-ARS   - Anti Jo-1 - Anti-PL7 - Anti-PL12 - Anti-EJ - Anti-OJ - Anti-KS | 18.5 (3.7 – 40.1)  ELISA, IP, IB/ELISA, DID-IIF-IP  14.8 (0.0 – 33.3)  DB-IIF-ELISA, LB, ELISA-LB,  IP, ELISA, DID-ELISA, IP-IPB-ELISA,  IP-DID, ID, IPB, LB-IP, LB-IPB, LB-WB, MR  1.3 (0.0 – 3.4)  LB, NR, IP, IPB, LB-IP, MR  1.6 (0.0 – 5.1)  IP, NR, IPB, LB, LB-IP, LB, MR  2.3 (0.0 – 5.9)  IP, LB, MR  1.7 (0.0 – 6.7)  IP, IPB, LB  0.8 (0.8 – 0.9)  IP | 23.9 (0.0 – 54.1)  ELISA, IP, ELISA-IP, DID-IIF-IP, IPB, LB  16.1 (0.0 – 48.5)  DID-IP, ELISA, FEI, ELISA-IP, ID, IP, LB, IPB, MR  7.9 (0.0 – 16.7)  ELISA-IP, ID, IP, LB  3.8 (0.0 – 9.1)  ELISA-IP, ID, IP, LB  6.5 (0.0 – 30.3)  ELISA-IP, ID, IP, LB  1.5 (0.0 – 3.0)  IP  8.1 (6.2 – 10.0)  ELISA-IP, IP | 9.1 (0.0 – 25.8)  DID-IIF, IP, IP-DID  7.1 (0.0 – 22.2)  ELISA-LB-IP, IP, ELISA, IP-DID,  IP-ELISA, LB, MR  0.6 (0.0 – 1.3)  ELISA-LB-IP, LB  5.5 (0.0 – 24.0)  ELISA-LB-IP, IP-DID, LB  0.2 (0.0 – 0.3)  ELISA-LB-IP, IP-DID  0.3  ELISA-LB-IP  0.2 (0.0 – 0.3)  IP-DID | 3.4  LB  4.7 (1.7 – 7.8)  LB, MR  2.2 (1.8 – 2.6)  ELISA, MR  1.7 (1.7 – 1.7)  LB, MR  ---  ---  --- | 0.015*  0.049*  0.017*  0.341*  0.063*  > 0.999**  0.065* |
| Anti-Mi-2 | 6.4 (0.0 – 19.1)  IP, DID-IIF-IP, ELISA, ID, LB-IP,  LB, LB-IPB, LB-WB, MR, NR | 6.4 (0.0 – 38.2)  IPB, DID-IIF-IP, ID, IP, WB, LB | 17.8 (0.0 – 60.0)  DID-IIF-IP, IP, IP-DID, LB | 3.0 (2.5 – 3.5)  LB, MR | 0.127* |
| Anti-MDA5/CADM140 | 12.7 (0.0 – 53.3)  ELISA-IB, ELISA-IP-IPB,  IP, IP-ELISA, LB | 17.7 (0.0 – 67.0)  ELISA, ID, IP, IPB, IP-ELISA,  IP-IB-ELISA, LB, LB-ELISA | 12.1 (7.1 – 15.4)  ELISA, IP, NR | --- | 0.658* |
| Anti-MJ/NXP2 | 9.0 (0.0 – 23.5)  IP, IP-ELISA, IP-ELISA-IPB, LB | 6.4 (0.0 – 16.0)  IPB, WB, IP, LB, | 17.5 (4.0 – 32.5)  IP, IP-DID-IPB, IP-IPB | --- | 0.023* |
| Anti-TIF1α/γ (p155/140) | 10.2 (0.0 – 36.4)  IP, ID, LB, NR | 13.7 (0.0 – 52.8)  ELISA, IPB, ID, WB, IP, LB | 24.1 (0.0 – 39.2)  IP, IP-DID-IPB | --- | 0.110* |
| Anti-HMGCR | 13.0 (0.0 – 45.2)  ELISA, IP, LBI | 9.6 (0.0 – 30.8)  ELISA, ELISA-IP, IP, NR | 6.0  ELISA-IP | --- | > 0.999** |
| Anti-SAE | 3.2 (1.1 – 7.5)  IP, ID, IPB | 1.7 (0.0 – 5.5)  IPB, IP, LB | 0.9 (0.0 – 4.0)  IP-DID, IP | --- | 0.163* |
| Anti-SRP | 5.3 (0.0 – 42.6)  LB, IP, DID-IIF-IP, IPB, LBI, LB-IP, MR | 6.1 (0.0 – 36.4)  IP, DID-IIF.IP, ID, IPB, LB | 4.3 (0.0 – 18.2)  DID-IIF-IP, IP, IP-DID, LB | 1.2 (0.0 – 2.5)  LB | 0.615* |
| Anti- cN1A | --- | --- | 72.0  ELISA-WB | 34.8  ELISA | --- |
| Anti-RNP   - Anti-U1RNP - Anti-U3RNP - Anti-U5RNP | 6.5 (0.6 – 12.4)  ELISA/CI, NR  4.9 (2.2 – 7.4)  LB, IP, IP-DID, PB  0.6 (0.0 – 1.9)  LB, IP  2.3  IP | 0.8  LB  5.2 (0.0 – 14.9)  DID, IPB, IP  ---  --- | ---  3.8 (0.0 – 12.1)  IP-DID, IP  ---  --- | ---  ---  ---  --- | ---  0.433*  ---  --- |
| Anti-Ro   - Anti-Ro52 - Anti-Ro60 | 7.9 (3.4 – 12.1)  ELISA, ELISA/CI, IP, IPB, LB-WB, NR  21.3 (0.5 – 32.7)  LB, IP, LB-IPB, LB-WB  22.7  IP | 12.7 (0.0 - 44.8)  DID-IP, ID, IP, LB, IPB  23.0 (0.0 – 50.8)  ELISA, LB  6.0  1P | 5.6 (5.4 – 6.1)  IP-DID  23.2 (19.5 – 29.0)  ELISA, IP-ELISA  13.8 (6.5 – 24.0)  IP | ---  15.0 (8.7 – 29.6)  ELISA, LB, MR  --- | 0.596*  0.899*  --- |
| Anti-La | 2.8 (1.2 – 5.4)  ELISA, IPB, NR | 2.1 (1.6 – 3.0)  DID-IP, ID, LB | 0.0 (0.0 – 0.0)  IP-DID | --- | 0.052* |
| Anti-PMScl   - Anti-PMScl75 - Anti-PMScl100 | 9.5 (0.0 – 31.2)  IP, LB, IP-DID, LB-WB  9.5  LB  4.2  LB | 1.1 (0.0 – 3.3)  ID, IP  6.1 (0.0 – 16.0)  ELISA-IP, LB  3.5 (1.8 – 4.5)  ELISA-IP, LB | 5.1 (0.0 – 13.1)  IP, IP-DID  ---  --- | 8.7  MR  21.7  LB  1.8  ELISA | 0.056*  ---  --- |
| Anti-Ku | 2.3 (0.0 – 6.3)  LB, NR, IP, LB-IPB | 4.3 (0.0 – 13.6)  IP, ID, LB | 0.1 (0.0 – 0.3)  IP-DID, IP | 3.4 (2.6 – 4.3)  LB, MR | 0.116* |
| Anti-Su | 3.4  IP | 5.8  IP | --- | --- | --- |

$\bar{\boldsymbol{x}}$**:** mean; **min:** minimum; **max:** maximum; **MSA:** Myositis Specific Autoantibodies; **IIM:** Idiopathic Inflammatory Myopathies; **DM:** Dermatomyositis; **PM:** Polymyositis; **IBM:** Inclusion Body Myositis; **IMNM:** Immune-Mediated Necrotizing Myopathy**; ARS:** Aminoacyl tRNA Synthetase; **MDA5:** Melanoma Differentiation-Associated Gene 5; **NXP:** Nuclear Matrix Protein; **TIF1γ/α:** Transcription Intermediary Factor 1γ/α; **HMGCR:** Hydroximethylglutaryl coenzyme A reductase; **SAE:** Small ubiquitine-like modifier Activating Enzyme; **SRP:** Signal Recognition Particle; **cN1A**: cytosolic 5’ nucleotidase 1A; **RNP:** ribonucleoprotein; **DID:** Ouchterlony Double Immunodifussion; **IIF:** Indirect Immunofluorescence; **IP:** Immunoprecipitation: **DB:** Dotblot; **ELISA:** Enzyme-Linked Immunosorbant Assay; **FEI:** Fluorescence Enzyme Immunoassay; **MR:** Medical Records; **IPB:** Immunoprecipitation-blotting; **NR:** No Reported; **WB:** Western Blot; **LBI:** Laser Bead Immunoassay; **IB:** Immunoblot; **CI:** Chemiluminiscence Immunoassay. ^&^Non-parametric tests as appropriate; *Asymptotic; **Exact Fisher test.
