# Supplementary material for: Geographical Latitude Remains as an Important Factor for the Prevalence of Some Myositis Autoantibodies: A Systematic Review
Source: Front Immunol. 2021 Apr 22;12:672008. doi: 10.3389/fimmu.2021.672008 (PMC8100663; doi:10.3389/fimmu.2021.672008)
Supplement: Supplementary file 4 [file Table_3.docx]

**Supplementary Table 3. Categorization of reported countries according to mean annual UV radiation in 2010.**

| Country (n) | UV radiation  $\bar{\boldsymbol{x}}$(min – max)* | WHO UVI category | UV level |
| --- | --- | --- | --- |
| United Kingdom (9) | 2.6 (1.0 – 4.9) | 1 | 2 |
| Sweden (6) | 2.7 (1.0 – 5.0) |  |  |
| Czech Republic (3) | 2.8 (1.0 – 5.0) |  |  |
| Netherlands (1) | 2.8 (1.0 – 5.0) |  |  |
| Germany (1) | 2.8 (1.0 – 5.0) |  |  |
| Canada (1) | 2.9 (1.0 – 7.0) |  |  |
| France (4) | 3.0 (1.0 – 5.7) | 2 | 3 |
| Poland (1) | 3.0 (1.0 – 6.0) |  |  |
| Hungary (5) | 3.1 (1.0 – 6.0) |  |  |
| Chile (1) | 3.3 (1.0 – 5.0) |  |  |
| South Korea (2) | 3.7 (1.0 – 7.0) |  |  |
| Japan (42) | 4.1 (1.6 – 6.9) |  | 4 |
| Italy (12) | 4.2 (2.1 – 6.3) |  |  |
| United States of America (19) | 4.3 (2.0 – 6.5) |  |  |
| Spain (7) | 4.3 (1.7 – 6.3) |  |  |
| Mexico (4) | 4.5 (3.5 – 5.5) |  |  |
| Guatemala (1) | 4.5 (4.0 – 6.0) |  |  |
| Australia (4) | 4.6 (3.0 – 6.7) |  |  |
| China (21) | 4.7 (2.7 – 7.0) |  |  |
| Argentina (4) | 4.8 (3.0 – 7.0) |  |  |
| Greece (1) | 5.2 (3.0 – 7.0) |  | 5 |
| India (6) | 6.6 (4.5 – 8.7) | 3 | 6 |

**n:** number of reports; **UV**: ultraviolet**; WHO:** World Health Organization; **UVI:** UV index. *The reported UV levels correspond to minimum and maximum averages reported per city.
